# CircFndc3b Mediates Exercise‐Induced Neuroprotection by Mitigating Microglial/Macrophage Pyroptosis via the ENO1/KLF2 Axis in Stroke Mice

**DOI:** 10.1002/advs.202403818

**Published:** 2024-10-28

**Authors:** Yun Zhao, Xiaofei He, Xiaofeng Yang, Zhongqiu Hong, Yin Xu, Jinghui Xu, Haiqing Zheng, Liying Zhang, Zejie Zuo, Xiquan Hu

**Affiliations:** ^1^ Department of Rehabilitation Medicine The Third Affiliated Hospital Sun Yat‐sen University 600 Tianhe Road Guangzhou Guangdong 510630 China; ^2^ Department of Rehabilitation Zhujiang Hospital Southern Medical University 253 Industrial Middle Road Guangzhou Guangdong 510282 China

**Keywords:** circFndc3b, exercise, ischemic stroke, microglia, neurological dysfunction

## Abstract

Circular RNA (circRNA) plays a pivotal role in regulating neurological damage post‐ischemic stroke. Previous researches demonstrated that exercise mitigates neurological dysfunction after ischemic stroke, yet the specific contributions of circRNAs to exercise‐induced neuroprotection remain unclear. This study reveals that mmu_circ_0001113 (circFndc3b) is markedly downregulated in the penumbral cortex of a mouse model subjected to middle cerebral artery occlusion (MCAO). However, exercise increased circFndc3b expression in microglia/macrophages, alleviating pyroptosis, reducing infarct volume, and enhancing neurological recovery in MCAO mice. Mechanistically, circFndc3b interacted with Enolase 1 (ENO1), facilitating ENO1's binding to the 3' Untranslated Region (3'UTR) of Krüppel‐like Factor 2 (Klf2) mRNA, thereby stabilizing Klf2 mRNA and increasing its protein expression, which suppressed NOD‐like Receptor Family Pyrin Domain Containing 3 (NLRP3) inflammasome‐mediated microglial/macrophage pyroptosis. Additionally, circFndc3b enhanced ENO1's interaction with the 3′UTR of Fused in Sarcoma (FUS) mRNA, leading to increased FUS protein levels and promoting circFndc3b cyclization. These results suggest that circFndc3b mediates exercise‐induced anti‐pyroptotic effects via the ENO1/Klf2 axis, and a circFndc3b/ENO1/FUS positive feedback loop may potentiate exercise's neuroprotective effects. This study unveils a novel mechanism underlying exercise‐induced neuroprotection in ischemic stroke and positions circFndc3b as a promising therapeutic target for stroke management, mimicking the beneficial effects of exercise.

## Graphical abstract

Exercise‐induced upregulation of circFndc3b in microglia/macrophages alleviates pyroptosis following MCAO. Mechanistically, circFndc3b promotes ENO1's binding to the 3’UTR of Klf2 mRNA, stabilizing the mRNA and increasing Klf2 protein expression, thereby suppressing NLRP3 inflammasome‐mediated pyroptosis. Additionally, circFndc3b enhances ENO1's interaction with the 3’UTR of FUS mRNA, elevating FUS protein levels and promoting circFndc3b cyclization, further amplifying the neuroprotective effects of exercise.

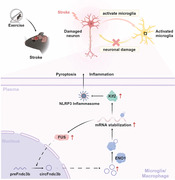

## 1 Introduction

Ischemic stroke, accounting for ≈80% of all stroke cases, presents a critical public health concern due to its high rates of morbidity, mortality, and disability.^[^ ^1^ ^]^ The underlying pathophysiology of ischemic stroke is multifaceted, often leading to ischemia‐reperfusion injury, predominantly driven by neuroinflammation.^[^ ^2^, ^3^ ^]^ As neuroinflammation significantly exacerbates ischemic brain damage, mitigating this process is essential for promoting neurological recovery following stroke.^[^ ^4^, ^5^ ^]^ Pyroptosis, a pro‐inflammatory form of cell death,^[^ ^6^ ^]^ further intensifies neuroinflammation and subsequent cerebral damage,^[^ ^7^ ^]^ with its activation in ischemic stroke primarily attributed to the NOD‐like Receptor Family Pyrin Domain Containing 3 (NLRP3) inflammasome,^[^ ^8^, ^9^ ^]^ particularly within microglia.^[^ ^10^, ^11^ ^]^ It is noteworthy that infiltrating circulating macrophages and resident microglia both play significant roles in the pathological processes of ischemic stroke, and distinguishing between these cells has been a technical challenge.^[^ ^12^ ^]^ Therefore, targeting both microglial and macrophage pyroptosis, and unraveling the mechanisms involved is essential for developing novel ischemic stroke therapies.

Exercise has gained recognition as a beneficial intervention in stroke rehabilitation, being associated with reduced incidence, severity, and mortality.^[^ ^13^ ^]^ Preclinical studies indicate that exercise prior to stroke reduces cerebral infarction and neuronal damage, with protective effects persisting for at least two weeks post‐stroke.^[^ ^14^ ^]^ Our previous study demonstrated that exercise attenuates neurological impairment in ischemic stroke through the regulation of neuroinflammation.^[^ ^15^ ^]^ Given that pyroptosis in microglia/macrophages is a critical driver of neuroinflammation, further exploration of exercise‐induced inhibition of pyroptosis in microglia/macrophages as a mechanism for alleviating neuroinflammation is warranted.

Circular RNA (circRNA), a novel class of non‐coding RNA characterized by its covalently closed‐loop structure lacking 5′ and 3′ ends, has emerged as a key player in neuroscience research.^[^ ^16^, ^17^ ^]^ Modulating circRNA expression offers promising therapeutic avenues for ischemic stroke.^[^ ^18^ ^]^ For instance, circSCMH1 has been shown to influence synaptic plasticity in peri‐infarct region and inhibit glial activation‐mediated neuroinflammation via interaction with transcription factor Methyl‐CpG‐binding protein 2.^[^ ^19^ ^]^ Nonetheless, the role of circRNA in regulating ischemic stroke pathophysiology remains incompletely understood. Several studies suggest that exercise regulates circRNA expression in various diseases. For example, circRNA levels correlate with exercise metrics, with circMBOAT2 serving as a potential biomarker for cardiopulmonary adaptability.^[^ ^20^ ^]^ CircRIMS2 potentially mitigatied vascular cognitive impairment via the miR‐186/Brain‐Derived Neurotrophic Factor axis in response to aerobic exercise.^[^ ^21^ ^]^ However, it remains unclear whether circRNA mediates the neuroprotective effects of exercise in the context of ischemic stroke.

Our study identified an exercise‐induced elevation of circFndc3b in the peri‐infarct cortex, where circFndc3b bound to Enolase 1 (ENO1), promoting Krüppel‐like Factor 2 (Klf2) expression and inhibiting NLRP3 inflammasome‐mediated microglial/macrophage pyroptosis. Moreover, circFndc3b enhanced Fused in Sarcoma (FUS) mRNA stabilization via ENO1, facilitating its cyclization. Consequently, exercise‐mediated circFndc3b exerted neuroprotective effects in ischemic stroke by regulating the circFndc3b/ENO1/Klf2 pathway and establishing a positive feedback loop via circFndc3b/ENO1/FUS, making circFndc3b a potential therapeutic target for stroke intervention.

## 2 Results

### 2.1 Exercise Effectively Attenuated Neuroinflammation and Microglial/Macrophage Pyroptosis, Conferring Neuroprotection in Middle Cerebral Artery Occlusion (MCAO) Mice

**Figure 1:**
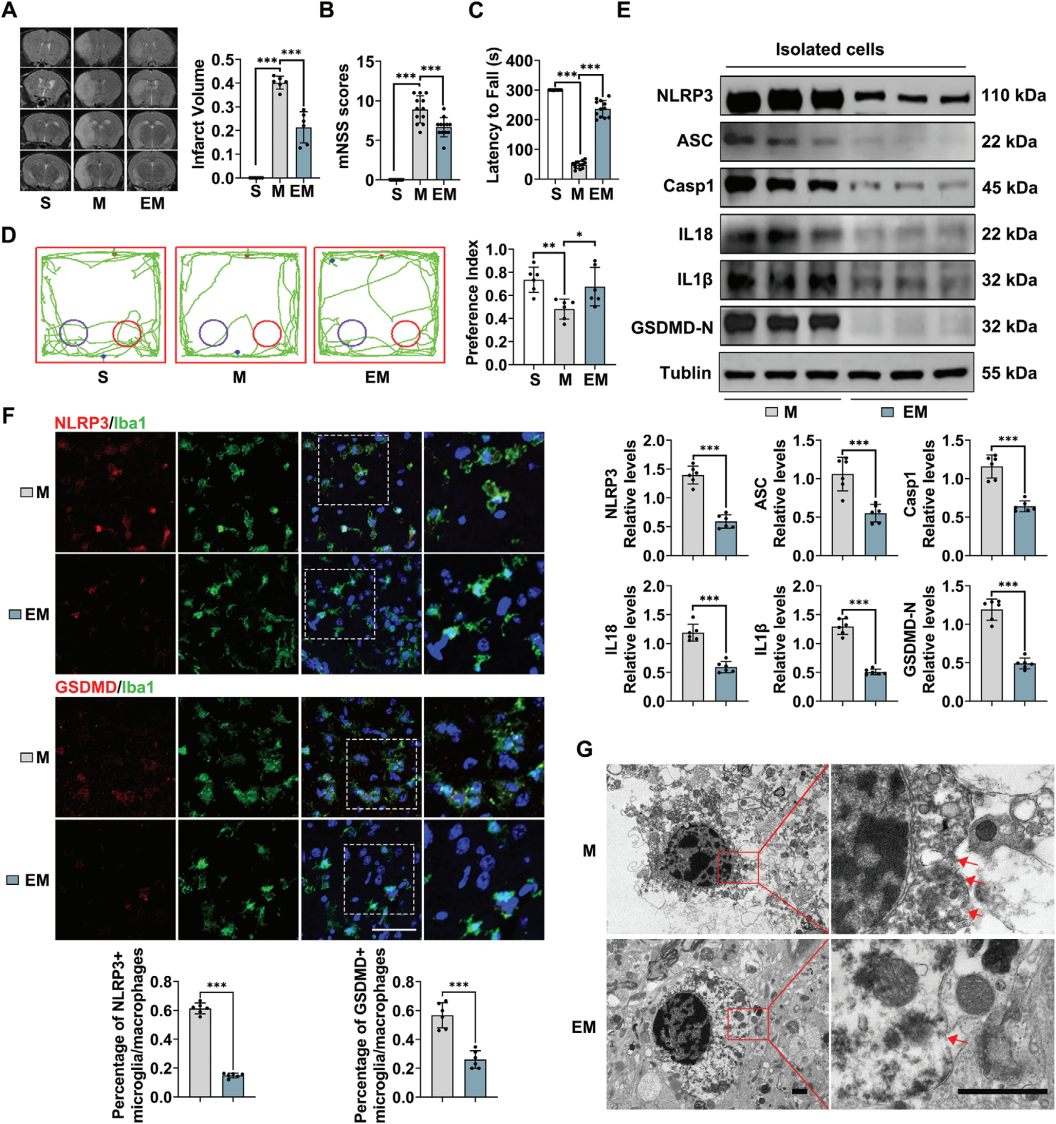
Exercise enhances neurological functional recovery following MCAO. (A) Representative MRI images and statistical analysis of infarct volume (n = 6). Neurological function was assessed using (B) mNSS (n = 12), (C) Rotarod test (n = 12), and (D) Novel Object Test (n = 6). (E) Western blot analysis of NLRP3, ASC, Casp1, IL18, IL1β, and GSDMD‐N expression in isolated microglia/macrophages from peri‐infarct cortex (n = 6). (F) Immunofluorescence staining of NLRP3/Iba1 and GSDMD/Iba1 in the peri‐infarct cortex (Scale bar = 50 µm), alongside quantitative analysis of NLRP3‐positive and GSDMD‐positive microglia/macrophages (n = 6). (G) Transmission electron microscopy images of microglia/macrophages in the peri‐infarct cortex (n = 3, Scale bar = 5 µm and 500 nm). Data are presented as mean ± SD. *P < 0.05, **P < 0.01, ***P < 0.001. For the statistical analysis of Figure A–D, a one‐way ANOVA followed by Tukey's post hoc test was conducted. For the statistical analysis of Figure E–F, an unpaired two‐tailed Student's *t*‐test was performed.

To evaluate the neuroprotective effects of exercise after MCAO, we conducted the experimental procedure as is shown in Figure S1A (Supporting Information). First, cerebral blood flow (CBF) data showed that ischemia caused a reduction in CBF to < 25% of baseline in both MCAO (M) and exercised (EM) groups, with recovery to 70%–80% thereafter (Figure S1B, Supporting Information), confirming consistent infarction levels between the groups. T2‐weighted imaging, conducted 24 h after MCAO, revealed a significant reduction in infarct volume in the EM group (*P* < 0.001, **Figure** **1**A) compared to the M group. Neurobehavioral assessments indicated improved performance in the EM group, as shown by improved mNSS scores (*P* < 0.001, Figure 1B), rotating rod test results (*P* < 0.001, Figure 1C), and preference index (*P* = 0.04, Figure 1D). These results suggest that exercise significantly attenuated neurological dysfunction in stroke mice.

Considering microglia/macrophage's central role in neuroinflammation, further investigation into exercise's impact on microglia/macrophage activation within the penumbra (Figure S1C, Supporting Information) was conducted. Immunofluorescence staining revealed a higher proportion of Arginase 1 (Arg1) positive microglia/macrophages and a lower proportion of Inducible Nitric Oxide Synthase (INOS) and Cluster of Differentiation 68 (CD68) positive microglia/macrophages in the EM group (*P* < 0.001, *P* = 0.001, *P* < 0.001, respectively, Figure S1D, Supporting Information), indicating that exercise reduced microglia/macrophage‐mediated neuroinflammation following ischemic stroke, consistent with previous findings.^[^ ^15^ ^]^ Morphometric analysis further showed that exercise increased microglia/macrophage's processed length and the number of branches in the penumbra compared to MCAO mice (both *P* < 0.001, Figure S1E, Supporting Information). The NLRP3 inflammasome pathway, a key player in microglial pyroptosis, was also examined. Western blot analysis of isolated microglia/macrophages from the penumbral cortex (Figure S2, Supporting Information) revealed significant reductions in NLRP3, Apoptosis‐associated Speck‐like Protein Containing a CARD (ASC), Cysteine‐aspartic Protease 1 (Casp1), Interleukin 18 (IL18), Interleukin 1 Beta (IL1β), and Gasdermin D (GSDMD) N‐terminal fragment (GSDMD‐N) levels in the EM group compared to the M group (all *P* < 0.001, Figure 1E). Immunofluorescence staining corroborated these findings, showing reduced percentages of GSDMD‐ and NLRP3‐positive microglia/macrophages in the penumbra of exercised mice (both *P* < 0.001, Figure 1F). Additionally, transmission electron microscopy (TEM) data indicated reduced microglia/macrophage edema and improved cellular integrity in the EM group (Figure 1G). Collectively, these results demonstrate that exercise effectively mitigates neuroinflammation and microglial/macrophage pyroptosis in the peri‐infarct region.

### 2.2 CircFndc3b Mediated the Neuroprotective Effects of Exercise via Inhibiting Neuroinflammation and Microglial/Macrophage Pyroptosis in MCAO Mice

**Figure 2:**
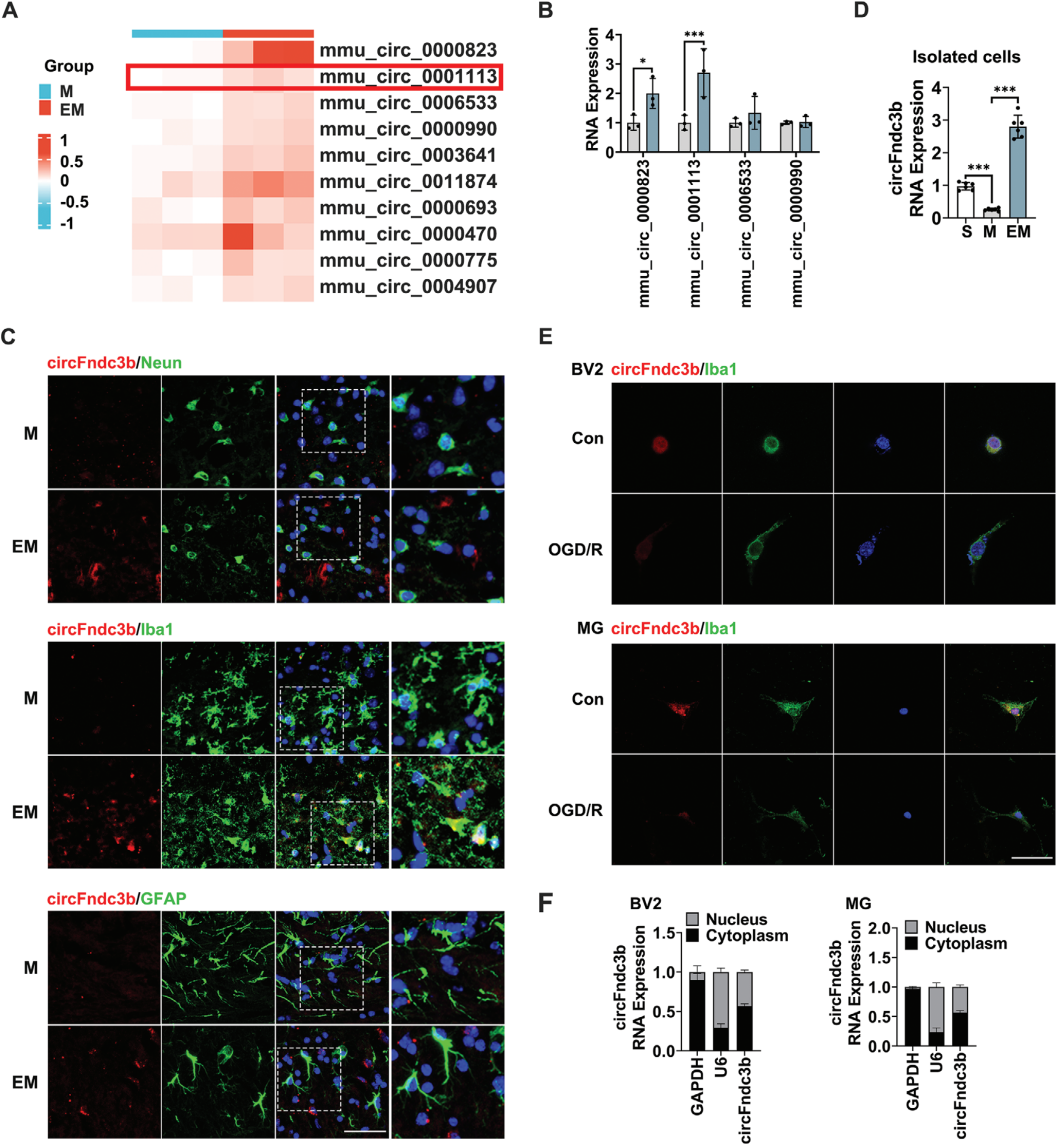
Exercise induces increased microglia/macrophage's circFndc3b expression in the peri‐infarct cortex after MCAO. (A) Heat map of differential circRNA expression in the peri‐infarct cortex after MCAO with or without exercise (n = 3). (B) Expression levels of selected circRNAs from circRNA‐seq data were validated by RT‐qPCR (n = 3). (C) RT‐qPCR quantification of circFndc3b expression in isolated microglia/macrophages from the peri‐infarct cortex across groups (n = 6). (D) Fluorescence in situ hybridization images using junction‐specific probes for circFndc3b, showing its distribution in neurons (Neun+), astrocytes (GFAP+), and microglia/macrophages (Iba1+) in the peri‐infarct cortex, as well as (E) its subcellular localization in BV2 and MG cells (n = 3, Scale bar = 50 µm). (F) RT‐qPCR quantification of circFndc3b in the cytoplasmic and nuclear fractions of BV2 and MG cells, with U6 and GAPDH as internal controls (n = 3). Data are presented as mean ± SD. *P < 0.05, ***P < 0.001. For the statistical analysis of Figure B, an unpaired two‐tailed Student's *t*‐test was performed. For the statistical analysis of Figure D, a one‐way ANOVA followed by Tukey's post hoc test was conducted.

To investigate the role of circRNAs in ischemic stroke and their potential mediation of exercise‐induced neuroprotection, circRNA sequencing was performed on the penumbral cortex of mice from the EM and M groups. Among the identified circRNAs (**Figure** **2**A, Table S1, Supporting Information), the top four upregulated candidates were validated by RT‐qPCR. Notably, mmu_circ_0 001113 (circFndc3b) was significantly upregulated in the exercised mice compared to the MCAO mice (*P* < 0.001, Figure 2B). RT‐qPCR and Sanger sequencing confirmed the cyclization site of circFndc3b (Figure S3A, Supporting Information), while agarose gel electrophoresis demonstrated that divergent primers amplified circFndc3b from complementary DNA but not genomic DNA (Figure S3B, Supporting Information). CircFndc3b expression showed a significant time‐dependent decrease after MCAO (all *P* < 0.001, Figure S4, Supporting Information). Fluorescence in situ hybridization (FISH) staining revealed that circFndc3b predominantly colocalized with Iba1‐positive microglia/macrophages (Figures 2C and S5, Supporting Information). In isolated microglia/macrophages from the penumbral cortex, circFndc3b was also notably higher at 24 h after MCAO in the EM group compared to the M group (*P* < 0.001, Figure 2D). In vitro, primary microglia (MG) were identified by their morphology and high Iba1 expression, confirming > 99% purity (Figure S6A,B, Supporting Information). After oxyglycan deprivation and reoxygenation (OGD/R), circFndc3b expression significantly decreased, with the lowest levels observed at 24 h in both BV2 and MG cells (all *P* < 0.001, Figure S7, Supporting Information). FISH staining after OGD/R also showed reduced circFndc3b fluorescence intensity in both BV2 and MG cells (Figure 2E). RNA nucleocytoplasmic isolation indicated that circFndc3b was present in both the nucleus and cytoplasm, with slightly higher levels in the cytoplasm (Figure 2F).

**Figure 3:**
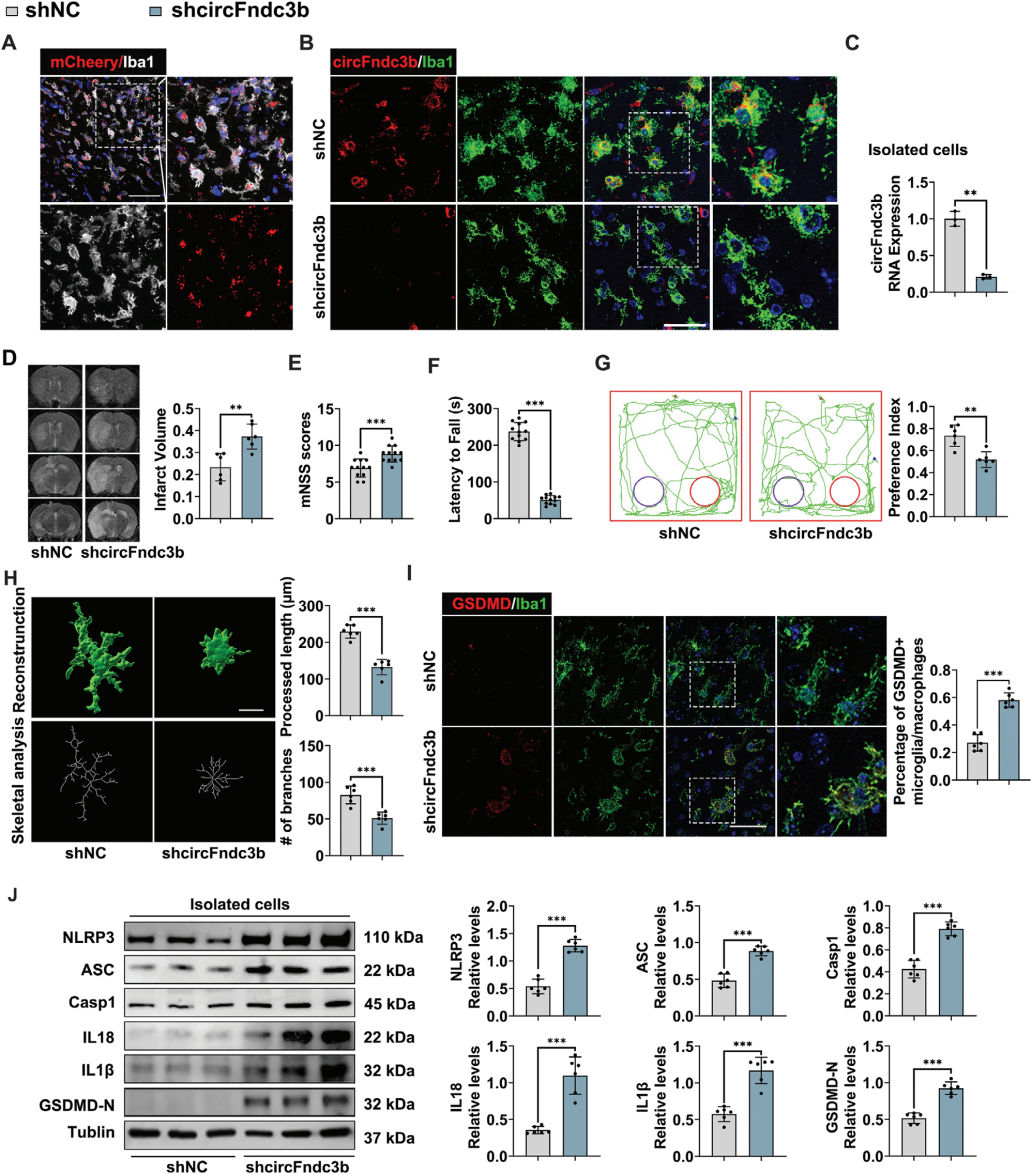
Silencing microglia/macrophage's circFndc3b partially reverses the neuroprotective effects of exercise after MCAO. (A) Representative immunofluorescence images of mCherry+ cells stained with Iba1 (n = 3, Scale bar = 50 µm). (B) Representative immunofluorescence images of Iba1+ cells stained with circFndc3b (n = 3, Scale bar = 50 µm). (C) RT‐qPCR quantification of circFndc3b expression in isolated microglia/macrophages from the peri‐infarct cortex across groups (n = 6). (D) Representative MRI images and statistical analysis of infarct volume (n = 6). Neurological function assessments were conducted using (E) mNSS (n = 12), (F) Rotarod test (n = 12), and (G) Novel Object Test (n = 6). (H) Imaris‐based 3D reconstruction images of microglia/macrophages immunofluorescently stained with Iba1, and the skeletal analysis of microglia/macrophages in the peri‐infarct cortex (n = 6, Scale bar = 20 µm). (I) Immunofluorescence staining of GSDMD/Iba1 in the peri‐infarct cortex (Scale bar = 50 µm), and quantitative analysis of GSDMD‐positive microglia/macrophages (n = 6). (J) Western blot analysis of NLRP3, ASC, Casp1, IL18, IL1β, and GSDMD‐N expression in isolated microglia/macrophages from the peri‐infarct cortex across groups (n = 6). Results are represented as means ± SD. *P < 0.05, **P < 0.01, ***P < 0.001. For the statistical analysis of Figure C–J, an unpaired two‐tailed Student's *t*‐test was performed.

To further investigate the role of microglia/macrophage's circFndc3b in mediating the neuroprotective effects of exercise after MCAO, rAAV (Recombinant Adeno‐associated Virus)‐SFFV‐DIO‐shRNA virus targeting either scramble (NC) or circFndc3b, with sh‐1 sequence chosen for its superior knockdown efficiency (Figure S8A,B and Table S2, Supporting Information), was delivered using AAV‐MG1.2 vectors into the left lateral ventricle of CX3CR1^CreERT2^‐EM mice 28 days before MCAO. Tamoxifen (75 mg kg^−1^) was administered intraperitoneally for five consecutive days to activate Cre recombinase and specifically downregulate microglia/macrophage's circFndc3b levels (Figure S8C, Supporting Information). Transfection efficiency was confirmed by the presence of mCherry‐tagged AAV, predominantly in microglia/macrophages, with minimal colocalization in neurons or astrocytes (**Figures** **3**A and S9, Supporting Information). This effectively reduced microglia/macrophage's circFndc3b expression (*P* < 0.001, Figure 3B,C). MRI scans revealed a significant increase in cerebral infarct volume in CX3CR1^CreERT2^‐EM mice treated with shcircFndc3b (*P* = 0.002, Figure 3D), as well as higher mNSS scores (*P* < 0.001, Figure 3E) and reduced rotarod latency (*P* < 0.001, Figure 3F). Additionally, a significant decrease preference index was observed in these mice (*P* = 0.001, Figure 3G). Morphometric analysis revealed that shcircFndc3b treatment reduced both the processed length and the number of branches of microglia/macrophages in the penumbra compared to shNC‐treated mice (both *P* < 0.001, Figure 3H). Immunofluorescence also indicated an increased percentage of GSDMD‐positive microglia/macrophages in the penumbral coretx (*P* < 0.001, Figure 3I). Western blot analysis showed upregulated expressions of NLRP3, ASC, Casp1, IL18, IL1β, and GSDMD‐N in isolated microglia/macrophages from the penumbral cortex in the shcircFndc3b‐treated group (all *P* < 0.001, Figure 3J). These results suggest that microglia/macrophage's circFndc3b knockdown impairs exercise‐induced neuroprotective and anti‐pyroptotic effects, highlighting its pivotal role in mediating these benefits.

### 2.3 CircFndc3b Inhibited Pyroptosis of Primary Microglia and BV2 Cells after OGD/R In Vitro

**Figure 4:**
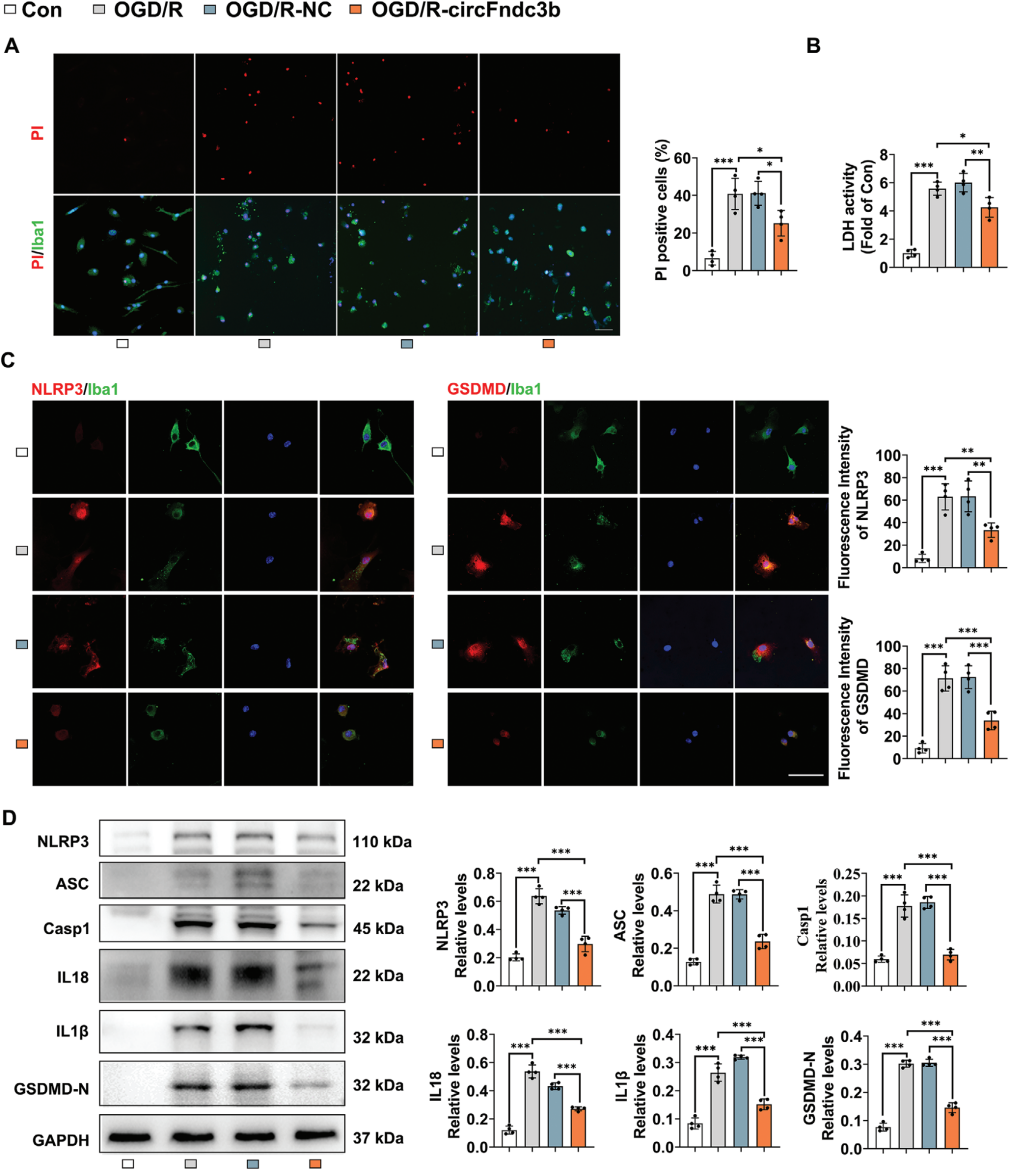
CircFndc3b overexpression in primary microglia mitigates NLRP3 inflammasome‐mediated pyroptosis after OGD/R. (A) Representative fluorescence images and quantification of PI‐positive cells in primary microglia overexpressing circFndc3b or NC following OGD/R (n = 4, Scale bar = 50 µm). (B) LDH activity in primary microglia overexpressing circFndc3b or NC after OGD/R (n = 4). (C) Representative immunofluorescence images of NLRP3/Iba1 and GSDMD/Iba1 staining in primary microglia overexpressing circFndc3b or NC after OGD/R (n = 4, Scale bar = 50 µm). (D) Western blot analysis of NLRP3, ASC, Casp1, IL18, IL1β, and GSDMD‐N expression in primary microglia overexpressing circFndc3b or NC after OGD/R (n = 4). Results are represented as means ± SD. *P < 0.05, **P < 0.01, ***P < 0.001. For the statistical analysis of Figure A–D, a one‐way ANOVA followed by Tukey's post hoc test was conducted.

Following OGD/R treatment in MG, a significant increase in cell death was observed (*P* < 0.001, **Figure** **4**A). However, overexpression of circFndc3b markedly attenuated this effect when compared to the OGD/R‐NC group (*P* = 0.021, Figure 4A). An lactate dehydrogenase (LDH) assay further demonstrated that circFndc3b overexpression significantly reduced LDH release, indicating its protective role against OGD/R‐induced cell death (*P* = 0.003, Figure 4B). Immunofluorescence staining revealed heightened NLRP3 and GSDMD fluorescence intensities in the OGD/R‐NC group, which were significantly diminished by circFndc3b overexpression (*P* = 0.004, *P* < 0.001, respectively, Figure 4C). Western blot analysis corroborated these findings, showing reduced expressions of NLRP3, Casp1, ASC, IL18, IL1β, and GSDMD‐N in circFndc3b overexpressing cells after OGD/R, indicating inhibition of NLRP3 inflammasome‐mediated pyroptosis and neuroinflammation (all *P* < 0.001, Figure 4D).

Similarly, BV2 cells subjected to OGD/R exhibited a significant rise in cell death 24 h post‐treatment (*P* < 0.001, Figure S10A, Supporting Information), which was further exacerbated by circFndc3b knockdown (*P* = 0.022 compared to the OGD/R‐shNC group). LDH assay results also showed increased LDH release following circFndc3b knockdown, indicating an aggravation of OGD/R‐induced cell death (*P* = 0.005, Figure S10B, Supporting Information). Immunofluorescence staining of NLRP3 and GSDMD in BV2 cells demonstrated elevated fluorescence intensities in the OGD/R groups, with even higher levels following circFndc3b knockdown (*P* = 0.027, *P* = 0.0375, respectively, Figure S10C, Supporting Information). Western blot analysis further confirmed that the OGD/R‐shcircFndc3b group exhibited significantly higher expressions of NLRP3, Casp1, ASC, IL18, IL1β, and GSDMD‐N compared to the OGD/R‐shNC group (*P* < 0.001, *P* = 0.0017, *P* = 0.0089, *P* < 0.001, *P* = 0.0127, *P* < 0.001, respectively, Figure S10D, Supporting Information). These results collectively suggest that circFndc3b plays a critical role in inhibiting pyroptosis in both MG and BV2 cells following OGD/R treatment.

### 2.4 CircFndc3b Bound to ENO1 and Does Not Affect the Expression Level of ENO1

**Figure 5:**
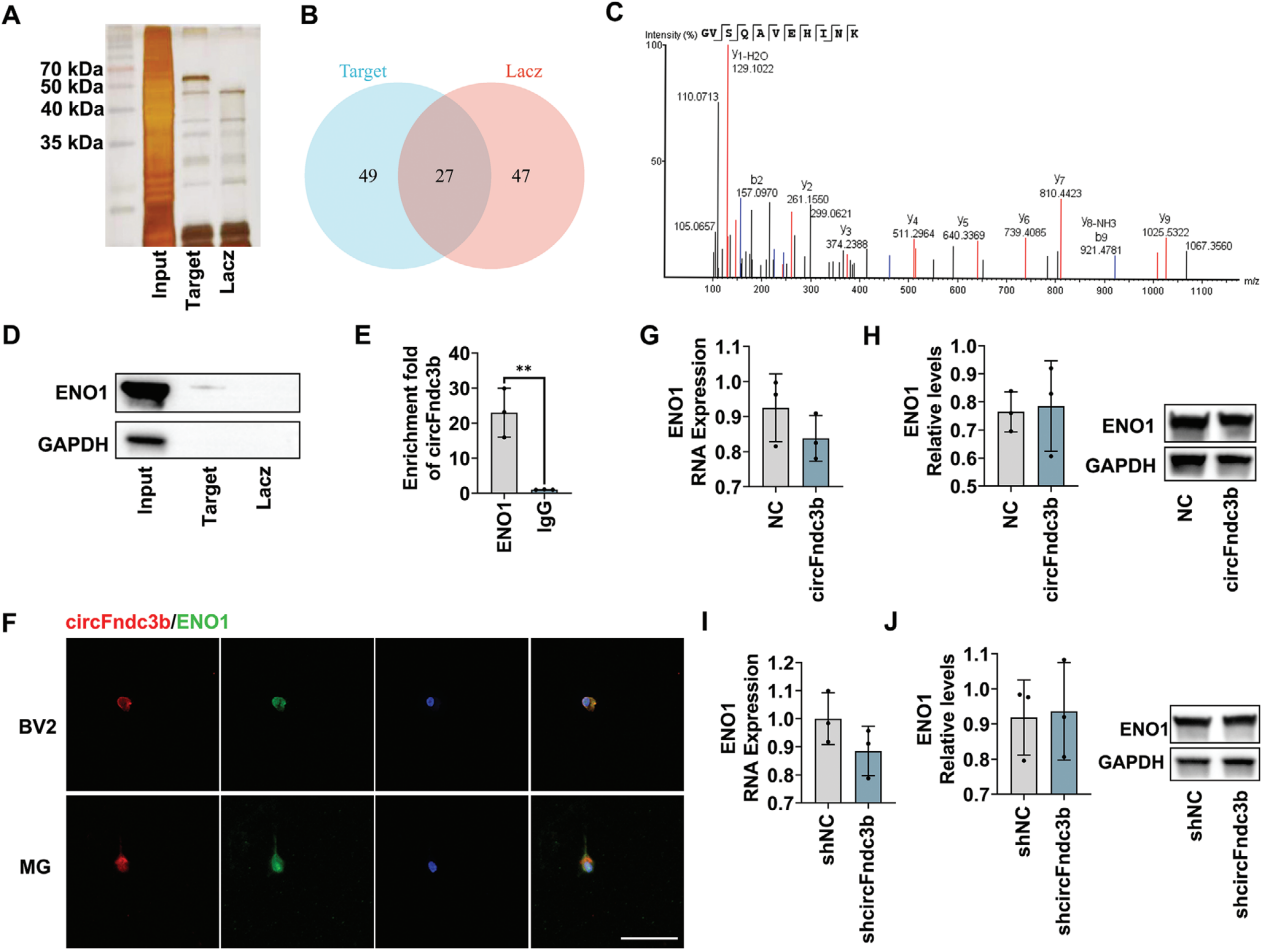
CircFndc3b interacts with ENO1 protein. (A) Representative silver staining images following RNA pulldown using biotinylated circFndc3b (Target) or negative control (Lacz) probes. (B) Schematic representation of proteins uniquely detected by LC‐MS with the circFndc3b probe. (C) LC‐MS identification of ENO1 as a circFndc3b‐binding protein. (D) RNA pulldown followed by western blot analysis confirmed circFndc3b binding to ENO1 (n = 3). (E) RNA immunoprecipitation using an ENO1 antibody, followed by RT‐qPCR analysis, showing circFndc3b enrichment in the ENO1 fraction (n = 3). (F) Co‐localization of circFndc3b and ENO1 detected by FISH and immunofluorescence, respectively (n = 3, Scale bar = 50 µm). (G) ENO1 mRNA levels (n = 3) and (H) ENO1 protein levels in BV2 cells overexpressing circFndc3b (circFndc3b) or NC (n = 3). (I) ENO1 mRNA levels (n = 3) and (J) ENO1 protein levels in BV2 cells with circFndc3b silencing (shcircFndc3b) or NC (shNC) (n = 3). Results are represented as means ± SD. *P < 0.05, **P < 0.01, ***P < 0.001. For the statistical analysis of Figure E, and G–J, an unpaired two‐tailed Student's *t*‐test was performed.

To further explore the functional role of circFndc3b, the expression of its parent gene, Fndc3b, was examined. Analysis of Fndc3b mRNA and protein levels in circFndc3b‐overexpressing MG and circFndc3b‐knockdown BV2 cells revealed no significant changes in Fndc3b expression (Figure S11, Supporting Information). Previous studies demonstrated circFndc3b's myocardial protective role in myocardial infarction, independent of the ceRNA mechanism.^[^ ^22^ ^]^ Given circRNA's diverse functions, it was hypothesized that circFndc3b might exert its effects via interactions with RNA‐binding proteins (RBPs). RNA pulldown followed by Western blotting identified distinct proteins between the circFndc3b probe and the negative Lacz probe (**Figure** **5**A). LC‐MS identified 49 unique proteins interacting with circFndc3b (Figure 5B, top 9 in Figure S12, Supporting Information, 49 unique proteins in Table S3, Supporting Information). ENO1, known to exacerbate neurological dysfunction when knocked down after ischemic stroke,^[^ ^23^, ^24^ ^]^ was of particular interest. ENO1, previously reported to act as an RBP regulating ferritin IRP1 mRNA stability,^[^ ^25^ ^]^ was predicted to interact with circFndc3b based on RPISeq^[^ ^26^ ^]^ and catRAPID^[^ ^27^ ^]^ models, particularly between ENO1 amino acids 176–227 and circFndc3b nucleotides 90–141 (Figure S13, Supporting Information). Thus, ENO1 was selected for further investigation (Figure 5C). Validation experiments, including RNA pulldown, RNA Immunoprecipitation (RIP), and immunofluorescence colocalization, confirmed the specific interaction between circFndc3b and ENO1 (Figure 5D–F). However, neither overexpression nor knockdown of circFndc3b affected ENO1 mRNA or protein levels in BV2 cells (Figure 5G–J).

### 2.5 CircFndc3b Promotes the Stabilizing Effect of ENO1 Protein on Klf2 mRNA

**Figure 6:**
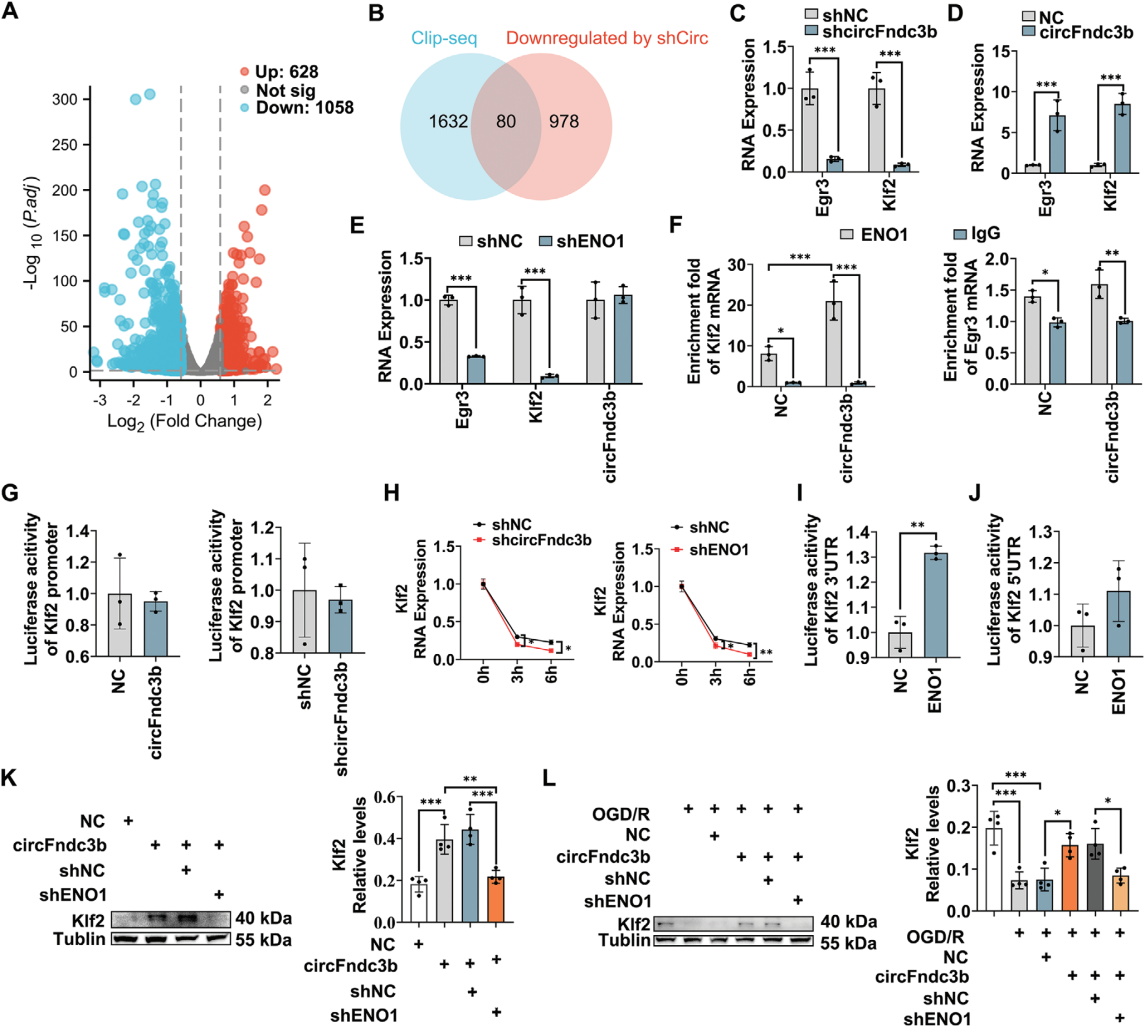
CircFndc3b enhances the binding of ENO1 to Klf2 mRNA. (A) Volcano plots showing differential mRNA expression in BV2 cells between control (shNC) and circFndc3b silencing (shcircFndc3b) groups (n = 3). (B) Schematic diagram illustrating the overlap of mRNA‐seq and ENO1 clip‐seq data to predict mRNAs with potential ENO1 binding. (C) Egr3 and Klf2 mRNA levels in BV2 cells with circFndc3b silencing or control (shNC) (n = 3). (D) Egr3 and Klf2 mRNA levels in BV2 cells overexpressing circFndc3b or control (NC) (n = 3). (E) Expression levels of Egr3, Klf2, and circFndc3b mRNA in shcircFndc3b and shNC groups (n = 3). (F) RNA immunoprecipitation using ENO1 antibody followed by RT‐qPCR showing enrichment of Klf2 and Egr3 mRNA in the ENO1 fraction (n = 3). (G) Luciferase reporter assay showing transcription levels of Klf2 in control and circFndc3b‐overexpressing or silencing groups (n = 3). (H) Klf2 mRNA stability in shNC, shcircFndc3b, or shENO1 groups was assessed by RT‐qPCR at 3 h and 6 h after ActD treatment (n = 3). (I) Luciferase reporter assay showing the activity of Klf2 3′UTR (n = 3), and (J) Klf2 5′UTR in control and ENO1‐overexpressing groups (n = 3). (K–L) Klf2 protein expression levels in each group (n = 4). Results are represented as means ± SD. *P < 0.05, **P < 0.01, ***P < 0.001. For the statistical analysis of Figure C–J, an unpaired two‐tailed Student's *t*‐test was performed. For the statistical analysis of Figure K–L, a one‐way ANOVA followed by Tukey's post hoc test was conducted.

To examine the regulatory effects of circFndc3b via ENO1, mRNA sequencing (mRNA‐seq) was performed on shNC and shcircFndc3b cells, followed by edgeR analysis^[^ ^28^ ^]^ (fold‐change ≥ 1.5, *P* < 0.05, FDR < 1). This revealed 628 upregulated and 1058 downregulated genes (**Figure** **6**A). By intersecting these genes with ENO1 CLIP‐seq data from RNAct,^[^ ^29^ ^]^ 80 downregulated genes were identified as ENO1 targets (Figure 6B, Table S4, Supporting Information). Among these, Early Growth Response 3 (Egr3) and Klf2 were significantly downregulated (≥ 5‐fold)^[^ ^30^ ^]^ in mRNA‐seq. Both were validated as downregulated in shcircFndc3b BV2 cells (both *P* < 0.001, Figure 6C), and upregulated in circFndc3b‐overexpressing BV2 cells (both *P* < 0.001, Figure 6D). RT‐qPCR in ENO1‐knockdown BV2 cells, with sh‐1 sequence chosen for its superior knockdown efficiency (*P* < 0.001, Figure S14, Table S2, Supporting Information), revealed no change in circFndc3b levels, but significant reductions in Egr3 and Klf2 mRNA levels (both *P* < 0.001, Figure 6E). RIP experiments further confirmed ENO1's enrichment of Egr3 and Klf2 mRNA, with increased Klf2 binding in circFndc3b‐overexpressing cells compared to cells in the NC group (*P* < 0.001 for Klf2 mRNA, *P* = 0.309 for Egr3 mRNA, Figure 6F), indicating that circFndc3b enhances ENO1's interaction with Klf2 mRNA. Using a Klf2 promoter Luciferase plasmid, no transcriptional impact of circFndc3b on Klf2 was observed (Figure 6G). An Actinomycin D (ActD) assay demonstrated significant reductions in Klf2 mRNA levels in shcircFndc3b (*P* = 0.0384 for 3 h, *P* = 0.0227 for 6 h) and shENO1 cells (*P* = 0.043 for 3 h, *P* = 0.007 for 6 h, Figure 6H), confirming their role in mRNA stability. A dual‐luciferase assay further revealed ENO1's binding to the 3′UTR rather than 5′UTR of Klf2 mRNA (*P* = 0.001, *P* = 0.184, respectively, Figure 6I,J). Rescue experiments indicated that circFndc3b overexpression significantly increased Klf2 protein levels, which was reversed by ENO1 knockdown (*P* < 0.001, Figure 6K). Following OGD/R, circFndc3b overexpression restored Klf2 protein levels compared to the shNC group (*P* = 0.015, Figure 6L), while ENO1 knockdown reduced them (*P* = 0.0196, Figure 6L). These results suggest that circFndc3b functions as a scaffold, promoting Klf2 mRNA stability and protein expression through its interaction with ENO1.

### 2.6 Exercise Inhibited Microglial/Macrophage Pyroptosis Through the circFndc3b/Klf2 Pathway after Ischemic Stroke

**Figure 7:**
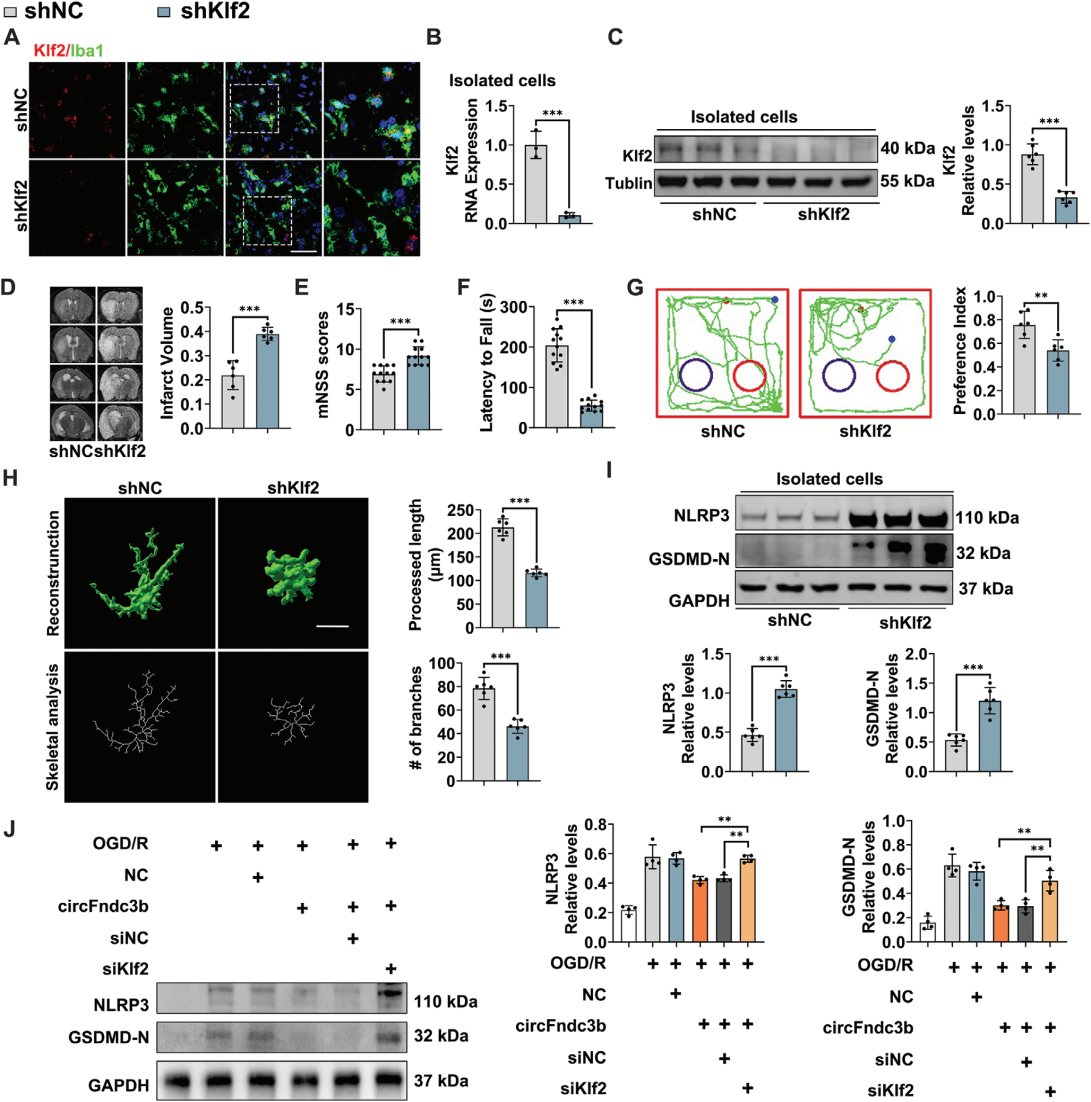
Microglia/maceophage's Klf2 mediates the neuroprotective effects of exercise after MCAO in a circFndc3b‐dependent manner. (A) Representative immunofluorescence images of Iba1+ cells stained with Klf2 (n = 3, Scale bar = 50 µm). (B) RT‐qPCR (n = 3) and (C) Western blot (n = 6) showing Klf2 shRNA silencing efficiency in isolated microglia/maceophages from the peri‐infarct cortex. (D) Representative MRI images and statistical analysis of infarct volume (n = 6). Neurological function assessments were performed using (E) mNSS (n = 12), (F) Rotarod test (n = 12), and (G) Novel Object Test (n = 6). (H) Imaris‐based 3D reconstruction images of microglia/macrophages immunofluorescently stained with Iba1, and the skeletal analysis of microglia/macrophages in the peri‐infarct cortex (n = 6, Scale bar = 20 µm). (I) Western blot analysis of NLRP3 and GSDMD‐N expression in the peri‐infarct microglia/macrophages (n = 6). (J) Western blot analysis of NLRP3 and GSDMD‐N expression in BV2 cells (n = 4). Results are represented as means ± SD. *P < 0.05, **P < 0.01, ***P < 0.001. For the statistical analysis of Figure B–I, an unpaired two‐tailed Student's *t*‐test was performed. For the statistical analysis of Figure J, a one‐way ANOVA followed by Tukey's post hoc test was conducted.

To determine whether microglia/macrophage's Klf2 mediates the anti‐neuroinflammatory and anti‐pyroptotic effects of exercise, Klf2 expression in isolated microglia/macrophages from the peri‐infarct cortex was first evaluated. As shown in Figure S15 (Supporting Information), exercise upregulated Klf2 mRNA and protein levels (*P* < 0.001 for both Klf2 mRNA and protein), while circFndc3b knockdown in exercised mice significantly decreased Klf2 levels (*P* < 0.001 for both Klf2 mRNA and protein), suggesting that exercise promotes Klf2 expression through circFndc3b. To further explore the neuroprotective role of microglia/macrophage's Klf2 in the exercised MCAO mice, rAAV‐SFFV‐DIO‐shRNA virus targeting Klf2, with si‐2 sequence chosen for its superior knockdown efficiency (*P* < 0.001, Figure S16A and Table S2, Supporting Information), was injected into the left lateral ventricle of CX3CR1^CreERT2^‐EM mice 28 days before MCAO, followed by tamoxifen (75 mg kg^−1^) administration for five consecutive days to induce Cre recombinase and downregulate microglia/macrophage's Klf2 (Figure S16B, Supporting Information). After MCAO, a significant decrease in microglia/macrophage's Klf2 expression was observed (**Figure** **7**A–C). RT‐qPCR and Western blotting further confirmed the downregulation of Klf2 following microglia/macrophage's Klf2 knockdown (both *P* < 0.001, Figure 7B,C). MRI scans after MCAO revealed a substantial increase in cerebral infarct volume in CX3CR1^CreERT2^‐EM mice treated with shKlf2 (*P* < 0.001, Figure 7D), along with higher mNSS scores (*P* < 0.001, Figure 7E) and reduced rotarod performance (*P* < 0.001, Figure 7F). Additionally, these mice showed a significant decrease preference index (*P* = 0.005, Figure 7G). Morphometric analysis further revealed that shKlf2 treatment reduced the processed length and the number of branches in microglia/macrophages around the penumbral cortex compared to the shNC group (both *P* < 0.001, Figure 7H). Western blot data demonstrated elevated NLRP3 and GSDMD‐N levels in CX3CR1^CreERT2^‐EM mice with shKlf2 treatment (both *P* < 0.001, Figure 7I), suggesting that microglia/macrophage's Klf2 plays a critical role in mediating exercise's neuroprotective and anti‐pyroptotic effects. In vitro rescue experiments indicated that Klf2 inhibition (siKlf2) after OGD/R reversed the suppression of NLRP3 and GSDMD‐N expression caused by circFndc3b overexpression (*P* = 0.0011, *P* = 0.0049, respectively, Figure 7J), confirming that circFndc3b exerts its neuroprotective effects via Klf2 upregulation following ischemic stroke.

### 2.7 FUS Regulated the Cyclization of circFndc3b

**Figure 8:**
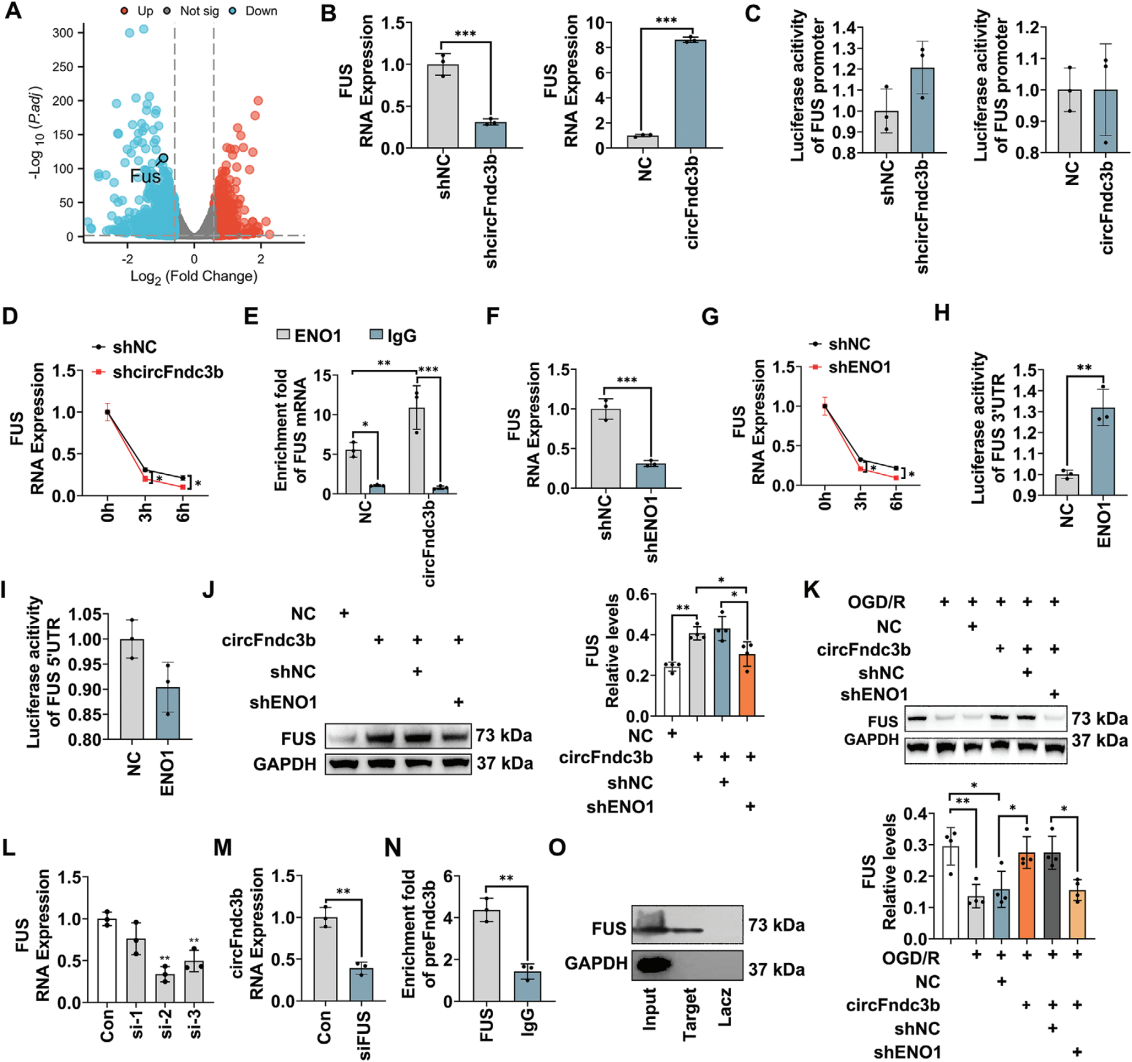
CircFndc3b, upregulated by FUS protein, stabilizes FUS mRNA by binding to ENO1 protein. (A) Volcano plots showing differential FUS gene expression between shNC and shcircFndc3b groups (n = 3). (B) FUS mRNA levels in circFndc3b overexpression, silencing, and control groups (n = 3). (C) Luciferase reporter assay demonstrating FUS transcription levels in circFndc3b overexpression, silencing, and control groups (n = 3). (D) FUS mRNA stability in shNC and shcircFndc3b groups assessed by RT‐qPCR at 3 h and 6 h post‐ActD treatment (n = 3). (E) RNA immunoprecipitation using ENO1 antibody followed by RT‐qPCR showing FUS mRNA enrichment in the ENO1 fraction (n = 3). (F) FUS mRNA levels in BV2 cells after ENO1 silencing or negative control (shNC) (n = 3). (G) FUS mRNA stability in shNC and shENO1 groups detected by RT‐qPCR after 3 h and 6 h ActD treatment (n = 3). (H) Luciferase reporter assay showing FUS 3′UTR activity (n = 3) and (I) FUS 5′UTR activity in control and ENO1‐overexpressing groups (n = 3). (J–K) FUS protein levels in each group (n = 4). (L) Silencing efficiency of FUS siRNA in BV2 cells (n = 3). (M) CircFndc3b levels in FUS‐silenced and control groups (n = 3). (N) RNA immunoprecipitation using FUS antibody followed by RT‐qPCR showing preFndc3b enrichment in the ENO1 fraction (n = 3). (O) RNA pulldown assay using biotinylated preFndc3b (Target) or negative control (Lacz), followed by western blot analysis showing preFndc3b bound to FUS protein (n = 3). Results are represented as means ± SD. *P < 0.05, **P < 0.01, ***P < 0.001. For the statistical analysis of Figure B–I and M–N, an unpaired two‐tailed Student's *t*‐test was performed. For the statistical analysis of Figure J–L, a one‐way ANOVA followed by Tukey's post hoc test was conducted.

Based on previous studies identifying circFndc3b's role in downregulating FUS expression in myocardial infarction,^[^ ^22^ ^]^ the relationship between circFndc3b and FUS was further explored. Our mRNA‐seq analysis revealed a significant reduction in FUS gene expression following circFndc3b knockdown (**Figure** **8**A), which was confirmed by RT‐qPCR, showing a notable decrease in the shcircFndc3b group compared to the shNC group (*P* < 0.001, Figure 8B). Conversely, circFndc3b overexpression markedly upregulated FUS mRNA levels (*P* < 0.001, Figure 8B). Using a FUS promoter Luciferase plasmid, no transcriptional impact of circFndc3b on FUS was observed (Figure 8C). The ActD assay demonstrated a significant decline in FUS mRNA expression in circFndc3b‐knockdown BV2 cells over time compared to the shNC group (*P* = 0.0477 for 3 h, *P* = 0.0457 for 6 h, Figure 8D). Given ENO1's role as an RBP,^[^ ^25^ ^]^ RIP experiments confirmed its direct interaction with FUS mRNA, with significant enrichment in circFndc3b‐overexpressing BV2 cells compared to the shNC group (*P* = 0.009, Figure 8E). ENO1 positively regulated FUS mRNA expression (*P* < 0.001, Figure 8F) and stability (*P* = 0.0365 for 3 h, *P* = 0.0296 for 6 h, Figure 8G). A dual‐luciferase assay further demonstrated that ENO1 binds to the FUS 3′UTR but not the 5′UTR (*P* = 0.003, *P* = 0.056, respectively, Figure 8H,I). Rescue experiments showed that ENO1 knockdown significantly reduced FUS expression in circFndc3b‐overexpressing BV2 cells (*P* = 0.015, Figure 8J). After OGD/R, circFndc3b overexpression upregulated FUS protein levels (*P* = 0.036, Figure 8K), but this effect was reversed by ENO1 knockdown (*P* = 0.031, Figure 8K). These results suggest that circFndc3b acts as a scaffold, enhancing ENO1 binding to FUS mRNA and promoting its stability and expression. Further studies have shown that FUS promotes circRNA cyclization^[^ ^31^, ^32^ ^]^ by binding near exon‐intron junctions in pre‐mRNA via GUGGU motifs. GUGGU sequences were identified near exons 2 and 3 of Fndc3b,^[^ ^33^ ^]^ suggesting FUS's involvement in circFndc3b cyclization. FUS knockdown experiments were first performed, and the si‐2 sequence was selected for subsequent experiments due to its most significant knockdown effect (*P* = 0.001, Figure 8L). Interestingly, in FUS‐knockdown BV2 cells, circFndc3b expression significantly decreased (*P* = 0.002, Figure 8M). RIP assay verified FUS protein binding directly to the pre‐mRNA of Fndc3b (preFndc3b) (*P* = 0.002, Figure 8N), and RNA pulldown using preFndc3b probe further confirmed this (Figure 8O), affirming FUS's regulatory role in circFndc3b cyclization.

## 3 Discussion

This study demonstrated that exercise markedly upregulated circFndc3b expression in microglia/macrophages within the peri‐infarct region, leading to the suppression of NLRP3 inflammasome‐driven microglial/macrophage pyroptosis and subsequent neuroinflammation, while also improving post‐stroke neurological deficits. The proposed mechanism involved the upregulation of circFndc3b, which promoted the binding of ENO1 to Klf2 mRNA, enhancing Klf2 mRNA stability and increasing its protein expression. This subsequently inhibited NLRP3 inflammasome‐mediated pyroptosis in microglia/macrophages, thereby improving the neuronal microenvironment and mitigating neurological dysfunction. Additionally, a self‐reinforcing circFndc3b/ENO1/FUS feedback loop was identified, offering insights into how exercise elevated circFndc3b levels in the peri‐infarct area. These results suggest that circFndc3b holds promise as a molecular target for reducing ischemic brain injury and represents a potential therapeutic strategy for ischemic stroke prevention and treatment.

Microglia/macrophages, the central nervous system's innate immune cells, are key mediators of neuroinflammation post‐ischemic stroke.^[^ ^34^, ^35^ ^]^ Within 24 h of stroke, damage‐associated molecular patterns activate microglia/macrophages, triggering microglial/macrophage pyroptosis, characterized by the formation of membrane pores, which exacerbates inflammation.^[^ ^36^, ^37^ ^]^ The NLRP3 inflammasome, a key pyroptosis inducer, is primarily activated in microglia,^[^ ^38^ ^]^ peaking at 24 h post‐stroke.^[^ ^36^ ^]^ Our findings corroborated this, showing that 24 h after MCAO, microglia/macrophages in the peri‐infarct cortex predominantly exhibited M1 markers (INOS/Iba1 and CD68/Iba1) and colocalized with NLRP3. GSDMD, the pyroptosis executioner,^[^ ^39^ ^]^ was also predominantly expressed in microglia/macrophages, indicating that NLRP3 inflammasome‐mediated pyroptosis was mainly localized to microglia/macrophages after stroke. Notably, exercise significantly reduced NLRP3 and GSDMD‐positive microglia/macrophages, indicating that exercise effectively downregulated NLRP3 inflammasome‐mediated microglial/macrophage pyroptosis. Transmission electron microscopy further confirmed characteristic pyroptotic features in peri‐infarct microglia, such as cellular swelling and enlarged membrane pores,^[^ ^40^ ^]^ which were notably attenuated by exercise. These results strongly underscore the neuroprotective effect of exercise in ischemic stroke, primarily through the reduction of microglial/macrophage pyroptosis.

CircRNA, a burgeoning area of research, is recognized for its multifaceted roles in various biological processes, with its modulation holding promise for novel ischemic stroke therapies.^[^ ^41^, ^42^, ^43^, ^44^ ^]^ Notably, research addressing the regulation of circRNA expression post‐ischemic stroke through exercise remains unknown, despite its substantial clinical implications. Our data revealed a significant reduction in circFndc3b levels in the peri‐infarct microglia/macrophages after MCAO, which was notably reversed by exercise. To date, only one study, conducted using a myocardial infarction mouse model, has investigated circFndc3b (mmu_circ_0 001113), demonstrating its cardioprotective effects.^[^ ^22^ ^]^ Its human analog, hsa_circ_0 001361, has been shown to alleviate oxidative stress and regulate the NF‐κB pathway in osteoarthritis through the miR‐525‐5p/HO‐1 axis, thus safeguarding chondrocyte extracellular matrix integrity.^[^ ^45^ ^]^ In our study, to elucidate the biological function of circFndc3b, a Cre‐LoxP‐based AAV‐shRNA system was utilized to selectively knock down microglia/macrophage's circFndc3b in the murine brain. Inhibition of microglia/macrophage's circFndc3b markedly negated the neuroprotective effects of exercise, intensifying microglial/macrophage pyroptosis and neuroinflammation. Additionally, in an in vitro OGD/R model simulating ischemic stroke, circFndc3b overexpression significantly mitigated NLRP3 inflammasome‐mediated microglial pyroptosis after OGD/R, while its knockdown exacerbated these effects. These complementary in vivo and in vitro findings established circFndc3b as a critical molecule in preserving neurological function during ischemic stroke and mediating the neuroprotective effects of exercise.

CircRNAs primarily exert their biological functions by competing for miRNA or RBP binding through the ceRNA mechanism.^[^ ^46^ ^]^ Previous research highlighted circFndc3b's cardioprotective function in myocardial infarction through interactions with RBPs rather than miRNAs.^[^ ^22^ ^]^ Based on circRNA's known mechanisms, we hypothesized that circFndc3b may interact with RBPs in this study. Our data revealed a slight predominance of circFndc3b in the cytoplasm compared to the nucleus. RNA pulldown, RIP, FISH, and immunofluorescence assays confirmed ENO1's specific binding to circFndc3b in the cytoplasm. Using the catRAPID algorithm, a potential interaction between ENO1 and circFndc3b was predicted, with the highest binding affinity predicted between ENO1 amino acids 176–227 and circFndc3b nucleotides 90–141. However, precise binding sites remain unconfirmed, necessitating further exploration in future studies.

ENO1 functions not only as a key glycolytic enzyme but also as an RBP that regulates mRNA stability,^[^ ^25^ ^]^ and as a DNA‐binding protein, it serves as a transcriptional repressor by inhibiting myc gene transcription.^[^ ^47^ ^]^ Previous research has shown that ENO1 knockdown worsened neurological deficits in ischemic stroke models.^[^ ^23^, ^24^ ^]^ Similar to other RBPs, including nucleolin and FUS, ENO1 directly binds to target mRNAs to regulate their stability.^[^ ^25^, ^48^, ^49^ ^]^ Our findings revealed that circFndc3b overexpression enhanced Klf2 mRNA stability, while knockdown of either circFndc3b or ENO1 reduced Klf2 expression. Moreover, the circFndc3b‐mediated upregulation of Klf2 levels was attenuated when ENO1 was knocked down. RIP experiments further demonstrated that circFndc3b overexpression increased the interaction between ENO1 and Klf2 mRNA, indicating that circFndc3b acts as a scaffold to facilitate ENO1 in recruiting and regulating the stability of Klf2 mRNA. Additionally, dual‐luciferase assays showed that ENO1 stabilized Klf2 mRNA by binding to its 3′UTR. A recent study revealed that ENO1, independent of its glycolytic activity, enhances YAP1 expression by binding to YAP1's 3′UTR in a YTHDF3‐dependent manner.^[^ ^50^ ^]^ YTHDF3, recognized for its role in binding m6A‐modified mRNAs, plays a pivotal role in modulating mRNA stabilization and translation efficiency.^[^ ^51^, ^52^ ^]^ Whether YTHDF3 or similar proteins are involved in ENO1's binding to Klf2's 3′UTR to stabilize Klf2 mRNA remains an open question and presents a valuable avenue for future research.

Klf2, a transcription factor, is crucial in various biological processes such as adipogenesis, lung development, and T‐cell survival.^[^ ^53^, ^54^ ^]^ Klf2 has shown protective effects in ischemic stroke, where it promotes post‐stroke angiogenesis^[^ ^55^ ^]^ and activates the IRF4/HDAC7 axis to reduce neuronal injury.^[^ ^56^ ^]^ However, its role in neuroinflammation following ischemic stroke remains unclear. In an endothelial inflammation model, Klf2 upregulation inhibited the NLRP3/Caspase‐1/IL‐1β pathway, reducing inflammation.^[^ ^57^ ^]^ It also mediated the expression of Forkhead Box Protein P1 in atherosclerosis‐prone endothelial cells induced by simvastatin, attenuating NLRP3 inflammasome activation and vascular inflammation.^[^ ^58^ ^]^ Additionally, Klf2 inhibited oxidized LDL‐induced NLRP3 inflammasome activation and endothelial cell pyroptosis.^[^ ^59^ ^]^ In this study, exercise significantly increased Klf2 expression in microglia/macrophages, an effect reversed by circFndc3b knockdown. Furthermore, Klf2 knockdown in microglia/macrophages markedly abrogated the neuroprotective benefits of exercise in vivo. In circFndc3b‐overexpressing microglia under OGD/R conditions, siRNA‐mediated Klf2 inhibition counteracted circFndc3b's suppressive effects on NLRP3 inflammasome activation, pyroptosis, and inflammation. Considering that circFndc3b acts as a scaffold for ENO1 to stabilize Klf2 mRNA, these results suggest that exercise may confer neuroprotection after ischemic stroke via the circFndc3b/ENO1/Klf2 pathway.

FUS, a member of the FET (FUS/EWS/TAF15) protein family, consisting of 526 amino acids,^[^ ^60^ ^]^ has been shown to facilitate circRNA cyclization through its interaction with pre‐mRNA.^[^ ^31^, ^61^, ^62^, ^63^, ^64^ ^]^ Primarily, it binds to pre‐mRNA introns near exon junctions,^[^ ^31^ ^]^ promoting circRNA formation by interacting with GUGGU sequences within introns.^[^ ^33^ ^]^ For example, FUS enhances circHIFlA formation by binding upstream of exon 2 in HIF1A pre‐mRNA, impacting breast cancer proliferation and metastasis.^[^ ^65^ ^]^ In the current study, circFndc3b is derived from back‐splicing between Fndc3b exons 2 and 3, where GUGGU sequences were identified in adjacent intronic regions, suggesting FUS may promote circFndc3b cyclization. FUS knockdown in BV2 cells reduced circFndc3b levels. RIP and RNA pulldown assays confirmed a direct interaction between FUS and preFndc3b, though specific binding sites remain to be identified. Moreover, knockdown of both circFndc3b and ENO1 led to a marked reduction in FUS expression. ActD and RIP experiments indicated that circFndc3b serves as a scaffold, facilitating ENO1's regulation of FUS mRNA stability. Furthermore, dual‐luciferase assays revealed that circFndc3b and ENO1 did not regulate FUS transcriptionally, but ENO1 specifically targeted the FUS 3′UTR. Overall, we proposed that FUS plays a pivotal role in circFndc3b cyclization, and a positive feedback loop involving circFndc3b/ENO1/FUS exists.

In summary, this study reveals the complex mechanisms through which exercise suppresses NLRP3 inflammasome‐driven microglial/macrophage pyroptosis and neuroinflammation, a process regulated by the circFndc3b/ENO1/Klf2 axis and reinforced by the circFndc3b/ENO1/FUS feedback loop. Collectively, these findings provide compelling evidence for circFndc3b as a promising biomarker for tracking neurological recovery post‐ischemic stroke and as a novel therapeutic target for replicating the neuroprotective effects of exercise, offering new hope for ischemic stroke treatment.

## 4 Experimental Section

### Animals and Exercise

Specific Pathogen‐Free (SPF) male C57BL/6J mice (Charles River, Beijing) and CX3CR1^CreERT2^ mice (Jinzhihe, Foshan), weighing 15–17 grams and aged 4–5 weeks, were utilized in this study. The mice were housed in an SPF facility at Guangzhou Laian Biological Co., Ltd., under controlled temperature, humidity, and a 12 h light‐dark cycle. Following the exercise protocol by He et al.,^[^ ^66^ ^]^ mice in the exercise group were placed in cages equipped with running wheels (diameter ≈17 cm) and had ad libitum access to food and water for four weeks. This study, conducted under license G2021015, adhered to ethical standards and was approved by Laian Technology and Biology Co., Ltd.’s Experimental Animal Ethics Committee.

### Transient Middle Cerebral Artery Occlusion

Transient Middle Cerebral Artery Occlusion (MCAO) was induced according to Shen et al.’s protocol.^[^ ^67^ ^]^ Anesthesia was administered using 3% isoflurane, maintained at 1.5%–2% during surgery. Mice were positioned supine, the cervical skin was prepared, and perivascular tissues were dissected with microtweezers. The left external carotid artery was ligated using a 6–0 silk suture at the internal‐external carotid bifurcation. A suture was inserted into the external carotid artery and advanced ≈9–10 mm to the junction of the middle and anterior cerebral arteries, securing it with silk thread. After 1 h of occlusion, the suture was withdrawn to restore blood flow. In the sham (f) group, the same surgical procedure was performed without occlusion. Following surgery, wounds were sutured and disinfected with iodophor. Subsequently, the mice were kept on a 37 °C warming pad with liquid food provided and then returned to their cages post‐recovery.

### Cerebral Blood Flow

Cerebral blood flow (CBF) in the cortical region was monitored using a Rayward Laser Speckle Blood Flow Imager (RWD, Shenzhen). After disinfecting the scalp with iodophor, an incision was made to expose the skull, and a camera was aligned with the anterior fontanelle to measure blood flow. CBF was recorded 15 min before surgery, immediately after the procedure, and following reperfusion. The ischemic core was defined as regions with 0%–20% baseline CBF, while the penumbra was identified as areas with 20%–30% baseline CBF.

### Neurological Assessment

Neurological performance after MCAO was evaluated using three assessments: the Modified Neurological Severity Score (mNSS), the Rotarod Test, and the Novel Object Recognition Test. The mNSS measures motor, sensory, reflex, and balance functions, with scores ranging from 0 (normal) to 18 (severe impairment). Mild injuries are classified with scores of 1–6, while moderate injuries score 7–12. The Rotarod Test conducted using equipment from Xinruan, Shanghai, involved placing mice on a rod that accelerated from 5 to 40 rpm over 300 s, recording the time to fall. In the Novel Object Recognition Test, two objects (A and B) were placed at opposite ends of a chamber, and the mice were introduced with their backs facing the objects, exploring for 5 min. Behavior was tracked using Super MAZE software (Xinruan, Shanghai). After a 60 min delay, object B was replaced with a novel object (C), distinct in shape, color, and size. Mice were reintroduced for another 5 min exploration, and data were captured using Super MAZE. A preference index was calculated as the ratio of time spent exploring the novel object (red) to the total time spent on both objects, providing insights into cognitive function. These assessments offered comprehensive evaluations of neurological outcomes following MCAO in mice.

### Infarction Volume Measurement

Infarct volume was evaluated 24 h after MCAO using a mouse MRI system (M3^TM^, Israel). Anesthesia was administered with 3% isoflurane, and a T2‐weighted MRI sequence (T2WI) was employed with the following parameters: repetition time = 3000 ms, echo time = 69.12 ms, 10 layers, 1 mm layer thickness, 1 mm spacing, 16‐echo chain length, and a 25×25 mm field of view. The MRI data were analyzed with ImageJ software, calculating infarct volume percentage as: [right brain volume – (left brain volume – left brain infarct volume)]/right brain volume.^[^ ^15^ ^]^

### Fluorescence In Situ Hybridization and Immunofluorescence Staining

Brain tissues were sectioned into 20‐µm slices. Following triple washes in 0.01M PBS, both the tissue slices and paraformaldehyde‐fixed microglia were permeabilized with 0.1% TritonX‐100 for 20 min, followed by blocking at room temperature for 1 h. Primary antibodies were diluted as follows: Iba1 (Abcam, ab283319, 1:200), NeuN (Abcam, ab104224, 1:200), GFAP (Cell Signaling Technology, #3670, 1:400), CD31 (R & D, AF3628, 1:200), GSDMD (Abclonal, A22523, 1:200), NLRP3 (Cell Signaling Technology, #15 101, 1:200), INOS (Abcam, ab178945, 1:200), Arg1 (Servicebio, GB11285, 1:100), CD68 (Abcam, ab53444, 1:200), Klf2 (Sabbiotech, 54228‐2, 1:200), and ENO1 (Abcam, ab155102, 1:200). These were applied and incubated overnight at 4 °C. After washing, fluorescent secondary antibodies (Abcam, 1:200) were added and incubated for 1 h at room temperature. Sections were mounted using an anti‐fluorescent quenching solution (with or without DAPI), sealed, and stored at 4 °C. Fluorescence microscopy (ZEISS, Leica, Germany; Nikon, Japan) was used to capture images. CircFndc3b localization in the peri‐infarct cortex and in microglia cultured in vitro was visualized using Cy3‐labeled probes (sequence: AATTGCCC + TCAACCTGCTACC + TTGGATGCAC + TGAAGTGTC), following the instructions for the frozen section RNA FISH kit and cell FISH kit (Genepharma, Shanghai).

For morphometric analysis of microglia/macrophages, brain tissues were sectioned into 40‐µm slices. Fluorescence microscopy (Nikon, Japan) was used to capture images with 1.5 µm steps in the Z‐direction.^[^ ^68^ ^]^ Imaris software was employed for 3D reconstruction of Iba1‐positive cells within the penumbral cortex. Skeletal analysis was conducted to assess microglia/macrophage's branches and processed length by ImageJ software.

### Adult Mouse Microglia/Macrophage Isolation and Flow Cytometry Examination

Brain dissociation was conducted using the Brain Dissociation Kit (RWD, Shenzhen) according to the manufacturer's protocol. Mice were euthanized via cervical dislocation, and brain tissues were dissected into small fragments before being transferred into a gentleMACS C tube (RWD, Shenzhen) containing the enzyme mixture. The tube was attached to the gentleMACS Octo Dissociator with Heaters (Miltenyi Biotec, #130‐096‐427) for 30 min to facilitate dissociation. Post‐dissociation, the resulting cell suspension was passed through a 70 µm nylon mesh and centrifuged to collect the cells. Afterward, cell debris and red blood cells were removed. Microglia/macrophages were then isolated using the Magnetic‐Activated Cell Sorting technique with CD11b MicroBeads (STEMCELL Technologies, #18 970). To prevent non‐specific binding, isolated microglia/macrophages were blocked with FcR Blocking Reagent for 10 min. Cells were subsequently stained with FITC anti‐mouse CD11b (Biolegend, 101 205) and APC/Fire^TM^ 750 anti‐mouse CD45 (Biolegend, 103 153) in the dark. The samples were analyzed using the BD FACSverse^TM^ flow cytometer (BD, USA).

### Western Blot

For protein analysis, gels were run at 300 mA for 90 min, with adjustments made depending on the protein molecular weight. After electrophoresis, PVDF membranes (Millipore, US) were blocked with 5% skim milk for 1 h, washed three times with TBST, and incubated overnight at 4 °C with the following primary antibodies: NLRP3 (Cell Signaling Technology, #15 101, 1:1000), Casp1 (Abclonal, A16792, 1:1000), ASC (Abclonal, A16672, 1:1000), IL1β (Proteintech, 16806‐1‐AP, 1:1000), IL18 (Proteintech, 10663‐1‐AP, 1:1000), GSDMD‐N (Abclonal, A22523, 1:1000), Klf2 (Sabbiotech, 54228‐2, 1:500), FUS (Cell Signaling Technology, #67840S, 1:1000), ENO1 (Abcam, ab155102, 1:1000), Fndc3b (Proteintech, 22605‐1‐AP, 1:1000), GAPDH (Servicebio, GB15004, 1:1000), Actin (Affinity, T0021, 1:1000), and Tublin (Affinity, AF7011, 1:1000). Afterward, membranes were washed with TBST and incubated with secondary antibodies (Abcam, 1:1000). Visualization was performed using a freshly prepared ECL developing solution (Omni, Shanghai).

### Transmission Electron Microscope

For transmission electron microscopy, tissues from the penumbral cortex were sectioned into 1×1×2 mm strips. Samples were fixed in 1% osmium tetroxide, dehydrated using a graded acetone series, and embedded in resin. Ultrathin sections (100 nm) were prepared using an ultramicrotome (UC7, Leica, Germany), stained with uranium hydrogen oxide acetate and lead citrate, and visualized under a Tecnai G2 Spirit transmission electron microscope (FEI, US).

### RNA Sequencing Analysis

For circRNA‐seq, brain tissues from the penumbra cortex of M and EM groups were placed in enzyme‐free 1.5 ml EP tubes, sealed, and sent to Guangzhou Geneseed Biology for RNA extraction and analysis. For mRNA‐seq, cell pellets from shNC and shcircFndc3b groups were prepared similarly and sent to Jiangsu Azenta Biology for further processing.

### RT‐qPCR

Brain tissue and cell precipitates were homogenized in Trizol, followed by centrifugation with chloroform at a ratio of 5:1 (Trizol: chloroform). The supernatant was combined with ice‐cold isopropanol and centrifuged, and the resulting pellet was washed with 75% ethanol and absolute ethanol before being dissolved in 20 µl enzyme‐free water. After eliminating genomic DNA, reverse transcription was performed using a kit (Novizan, Shanghai), and gene expression was quantified by qPCR using the ^2‐ △△^ Ct method, where ^△^ Ct represents the difference between the Ct value of the target gene and the reference gene. The sequences of primers used in this study were listed in Table S5 (Supporting Information).

### Genomic DNA (gDNA) Extraction and Agarose Gel Electrophoresis

GDNA was extracted using the TIANamp Genomic DNA Kit (Tiangen Biochemical Company, Beijing). DNA templates from total RNA extraction and the reverse transcription kit (Vazyme, Shanghai) were subjected to agarose gel electrophoresis with primers for circFndc3b and GAPDH. The gels were exposed to UV light for visualization and photographed.

### Cell Culture

Primary mouse MG were isolated from P1‐P2 C57BL/6J mice following previously established protocols.^[^ ^69^ ^]^ Cortical tissue was sterilized in 75% ethanol, minced, and digested with trypsin‐EDTA. The digestion was halted using 5% FBS‐DMEM/F12, and cells were centrifuged and resuspended in DMEM/F12 supplemented with 10% FBS. Cells were cultured in T75 flasks at 37 °C with 5% CO2. After 14 days, microglia were harvested by shaking the flasks and seeded in 24‐well plates. Primary microglia were identified through immunofluorescence staining with anti‐Iba1. BV2 and HEK293FT cells were cultured in DMEM containing 10% FBS and 1% penicillin‐streptomycin.

### Actinomycin D Experiment

BV2 cells, at 60%–80% confluence in 6‐well plates, were treated with actinomycin D (MedChemExpress, US; final concentration 10 µg ml^−1^).^[^ ^70^ ^]^ RNA was extracted at 0, 3, and 6 h post‐treatment, and Klf2 and FUS expression levels were analyzed by RT‐qPCR.

### SiRNA Transfection

For gene silencing, siRNA targeting FUS and Klf2 was transfected into primary microglia and BV2 cells using MaxFect Lipofectamine 3000 (Geneze, Qidong). Cells were seeded in 6‐well plates two days prior to transfection. For the transfection, Solution A (MaxFect Lipofectamine 3000 in serum‐free Opti‐MEM) and Solution B (P3000 Reagent, serum‐free Opti‐MEM, and siRNA) were prepared, mixed, and incubated. The resulting mixture was added to the cells and incubated for 6–8 h, after which the medium was replaced with fresh DMEM. Gene expression was assessed via RT‐qPCR 48–72 h post‐transfection. The sequences of siRNA used in this study were listed in Table S2 (Supporting Information).

### AAV Injection

Four weeks prior to MCAO, mice received AAV injections as described previously.^[^ ^67^ ^]^ Mice were anesthetized with 3% isoflurane and positioned on a brain stereotaxic apparatus. After iodine disinfection and a scalp incision, stereotaxic coordinates were recorded. For targeting the left lateral ventricle: 0.35 mm posterior, 1.0 mm lateral to the bregma, and 2 mm in depth from the skull. A 2 µL volume of AAV was injected at a rate of 0.5 µL min^−1^. The needle remained in place for 10 min post‐injection before withdrawal, and the incision was sealed with bone wax followed by suturing. For selective inhibition of circFndc3b and Klf2 levels in microglia/macrophages, a Cre‐LoxP‐based AAV‐shRNA system was introduced. Recombinant AAV‐SFFV‐DIO‐shRNA vectors using AAV‐MG1.2,^[^ ^71^ ^]^ targeting scramble (NC), circFndc3b, and K1f2, were custom‐designed by Wuhan Shumi Biological Co., Ltd., and administered to CX3CR1^CreERT2^ mice.

### Oxyglycan Deprivation and Reoxygenation

Primary microglia and BV2 cells, at 60%–80% confluence, were subjected to oxyglycan deprivation and reoxygenation (OGD/R). The cells were incubated in glucose‐free DMEM in a hypoxic chamber (94% N2, 5% CO2, 1% O2) for 6 h, followed by reoxygenation in high‐glucose DMEM. Samples were collected at various time points (3, 6, 12, 24, 48, and 72 h) for subsequent analyses.

### RNA Nucleocytoplasmic Separation Assay

Total RNA from the cytoplasmic and nuclear fractions of BV2 and primary microglia was isolated using the RNA Nucleocytoplasm Isolation Kit (Norgen, Canada). Cell lysates were treated with Lysis Buffer J and centrifuged to separate RNA fractions. RNA concentrations were quantified, and RT‐qPCR was immediately performed.

### Virus Infection

Lentiviral vectors overexpressing or silencing circFndc3b and ENO1 were introduced into primary microglia and BV2 cells at an MOI of 20–40, with polybrene. After infection, cells were cultured in a fresh medium and selected using puromycin. The sequences of shRNA used in this study were listed in Table S2 (Supporting Information).

### Lactate Dehydrogenase Release Test

For lactate dehydrogenase (LDH) release assays after OGD/R, primary microglia and BV2 cells were seeded in 96‐well plates. LDH release was measured using a kit (Beyotime, Shanghai) after 1 h of incubation with the LDH‐releasing medium. Samples were centrifuged, and absorbance was measured at 490 nm. LDH release was calculated as: (absorbance of the experimental sample – absorbance of the control sample)/ (absorbance of maximum enzyme activity – absorbance of the control sample).

### PI Staining

After OGD/R, primary microglia and BV2 cells were seeded in 24‐well plates and treated with PI working solution (33.4 µg ml^−1^, Beyotime, Shanghai). After 15 min at 37 °C, cells were washed with PBS and mounted in an anti‐fade solution with DAPI. Fluorescence imaging was performed using a fluorescence microscope (Nikon, Japan) under light‐protected conditions.

### RNA Pull‐Down

Using a mmu_circ_0 001113 pulldown probe (Genepharma, Shanghai) and Fndc3b pre‐mRNA (preFndc3b) probe (Saicheng, Guangzhou), RNA pulldown assays were performed on BV2 cells. After cell lysis, the supernatant was incubated with the probe and rotated in a 37 °C oven. Following magnetic bead incubation, the solution was washed, followed by RNA and protein extraction. Proteins were analyzed via gel electrophoresis, silver staining, LC‐MS and western blot.

### LC‐MS

LC‐MS analysis, carried out by Guangzhou Saicheng Biotechnology Co., Ltd., involved enzymatic digestion of protein bands stained with silver dye, followed by LC‐MS and database searches for result interpretation.

### Dual‐Luciferase Assay

HEK293FT cells were transfected with plasmids to either overexpress or silence circFndc3b or ENO1 levels, alongside a PGL3‐luciferase plasmid encoding the 3′UTR, 5′UTR, and promoter regions of FUS and Klf2 (Saicheng, Guangzhou). Firefly and Renilla luciferase activities were quantified using the Dual‐luciferase assay system (Promega, US), with Renilla luciferase serving as the internal control. All assays were conducted in triplicate.

### RNA Immunoprecipitation

RNA complexes were precipitated using ENO1 (Abcam, ab155102, 5 µg) and FUS (Cell Signaling Technology, #67840S, 5 µg) antibodies, and target RNA was detected via RT‐qPCR. The RNA Immunoprecipitation (RIP) assays adhered to the protocol specified by the kit manufacturer (Geneseed, Guangzhou).

### Statistical Analysis

Statistical analyses were performed using GraphPad Prism 8.3 (San Diego, CA), with data presented as mean ± standard deviation (SD). Differences between two groups were analyzed with an unpaired two‐tailed Student's *t*‐test. For comparisons involving multiple groups one‐way ANOVA followed by Tukey's post hoc test was employed. Statistical significance was set at *P* < 0.05.

## Conflict of Interest

The authors declare no conflict of interest.

## Author Contributions

Y.Z., X.H., and X.Y. contributed equally to this study. X.H., L.Z., and Z.Z. performed conceptualization, supervision, and wrote, reviewed and edited. X.H., L.Z., X.H., and Z.Z. performed funding acquisition. Y.Z., X.H., X.Y., and Y.X. performed conceptualization, methodology, project administration, data curation, wrote the original draft, investigation, and validation. Z.H. performed formal analysis. J.X. and H.Z. wrote, reviewed and edited.

## Supporting information



Supporting Information

## Data Availability

The data that support the findings of this study are available on reasonable request from the corresponding author.
