# Supplementary material for: CircFndc3b Mediates Exercise‐Induced Neuroprotection by Mitigating Microglial/Macrophage Pyroptosis via the ENO1/KLF2 Axis in Stroke Mice
Source: Adv Sci (Weinh). 2024 Oct 28;12(1):2403818. doi: 10.1002/advs.202403818 (PMC11714177; doi:10.1002/advs.202403818)
Supplement: Supplementary file 1 — Supporting Information [file ADVS-12-2403818-s001.docx]

**Supporting Information**

**CircFndc3b Mediates Exercise-Induced Neuroprotection by Mitigating Microglial/Macrophage Pyroptosis via the ENO1/KLF2 Axis in Stroke Mice**

Yun Zhao^1, 2#^, Xiaofei He^1#^, Xiaofeng Yang^1#^, Zhongqiu Hong^1^, Yin Xu^2^, Jinghui Xu^1^, HaiQing Zheng^1^, Liying Zhang^1*^, Zejie Zuo^1*^, Xiquan Hu^1*^

Supplementary Figures and Figure legends


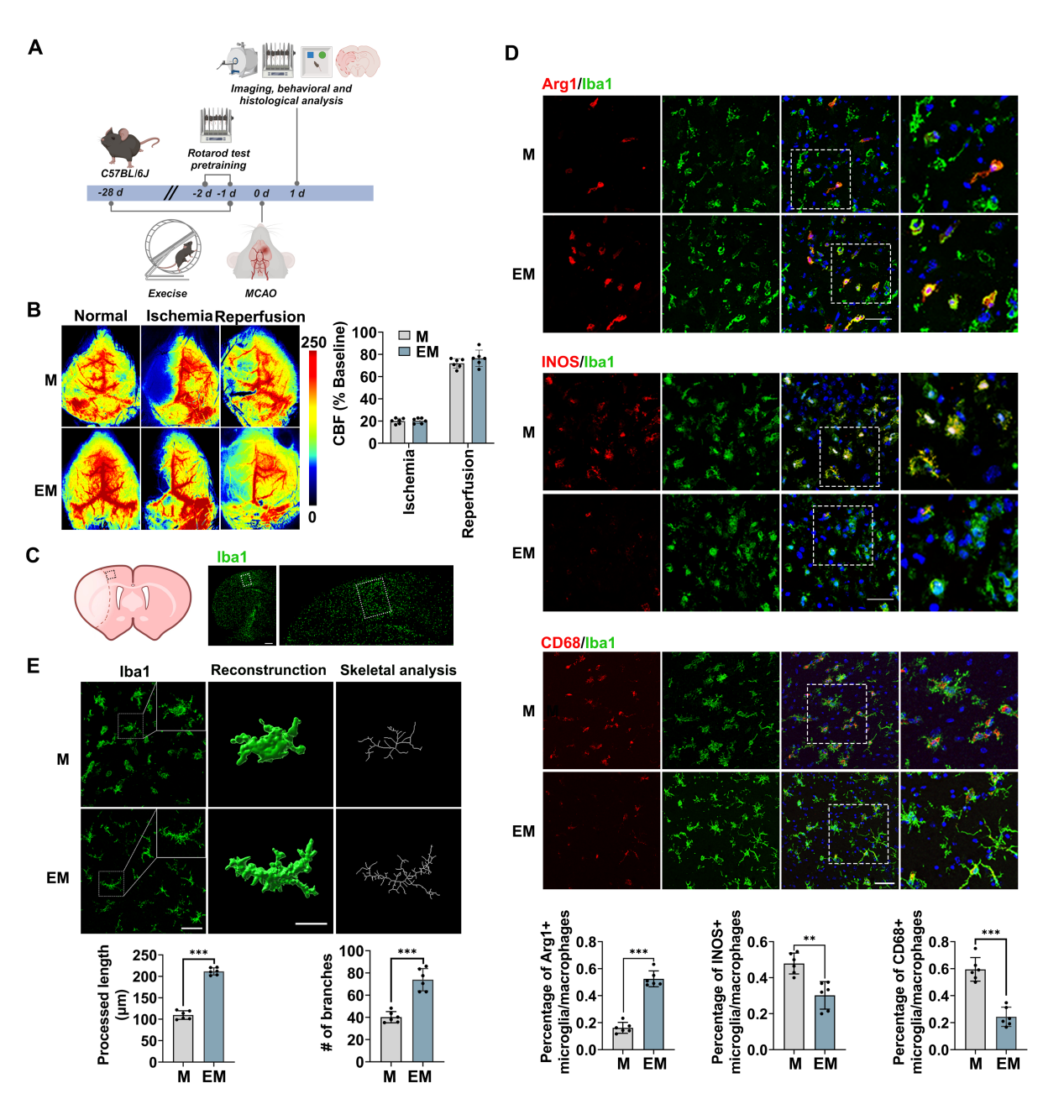


Figure S1. Exercise improved neuroinflammation-mediated by microglia/macrophages after MCAO.

1. Schematic illustration of the experimental procedures, created with Biorender. (B) Representative images of regional CBF, and quantification of CBF measured by laser speckle contrast imaging (n = 6). (C) Schematic illustration of the penumbra region (Scale bar = 500 μm). (D) Representative images of Arg1/Iba1, INOS/Iba1 and CD68/Iba1 staining in the peri-infarct cortex (Scale bar = 50 μm), and quantitative analysis of Arg1-positive, INOS-positive and CD68-positive microglia/macrophages (n = 6). (E) Imaris-based 3D reconstruction images of microglia/macrophages immunofluorescently stained with Iba1, and the skeletal analysis of microglia/macrophages in the peri-infarct cortex (n = 6, Scale bar = 20 μm). Results are represented as means ± SD. **P* < 0.05, ***P* < 0.01, ****P* < 0.001. For the statistical analysis of Figure D–E, an unpaired two-tailed Student’s t-test was performed.


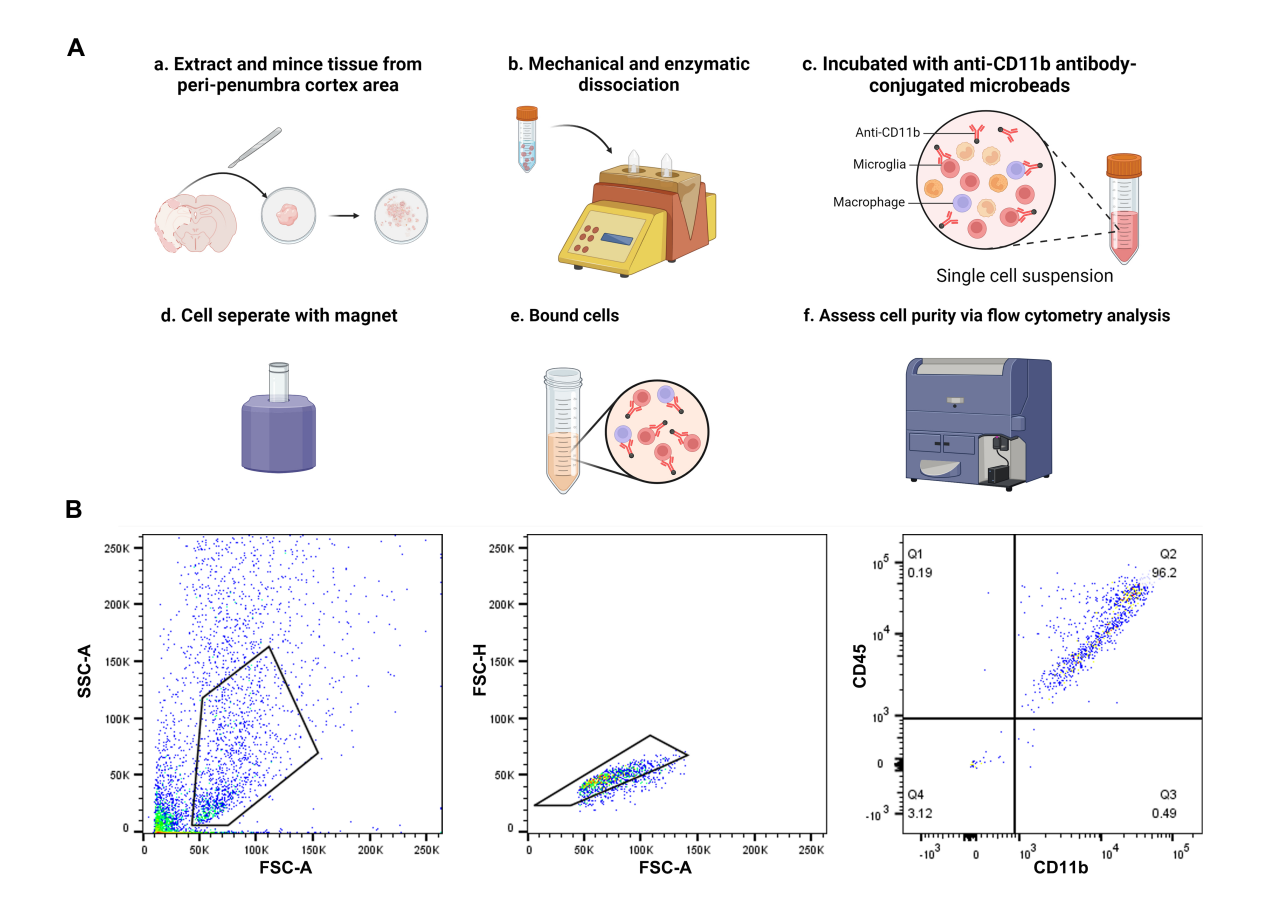


Figure S2. Adult mouse microglia/macrophage isolation from the penumbral cortex and flow cytometry examination.

(A) Schematic illustration of the experimental procedures for mouse microglia/macrophage isolation, created with Biorender. (B) Representative flow cytometry plots of gating strategy for CD11b+CD45+ microglia/macrophages population in the penumbral cortex.


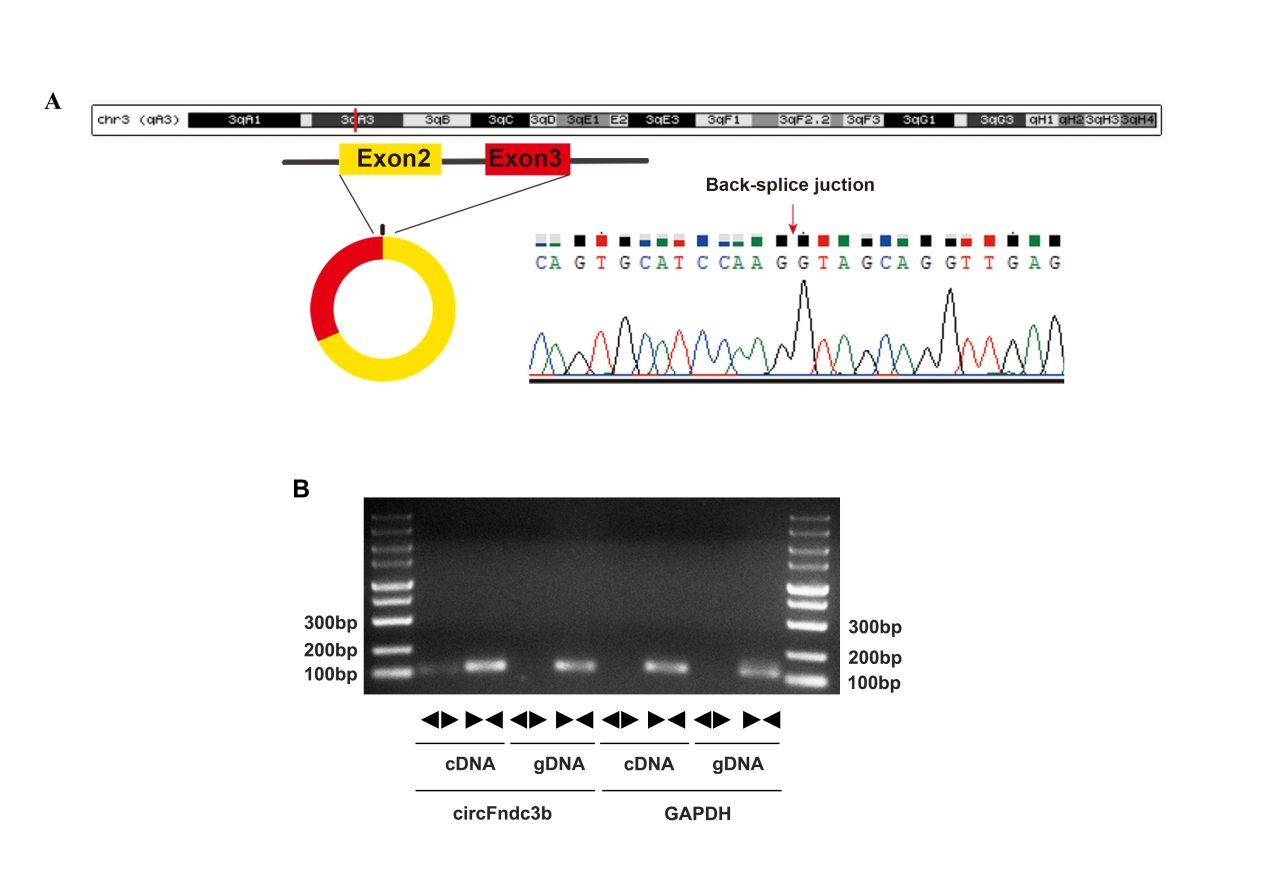


Figure S3. Expression of circFndc3b is present in the mouse brain tissue.

1. The exon composition of circFndc3b and the divergent primer of circFndc3b contains the back-splicing site. (B) Divergent primers amplified circFndc3b from cDNA, but not gDNA. GAPDH is used for linear control.


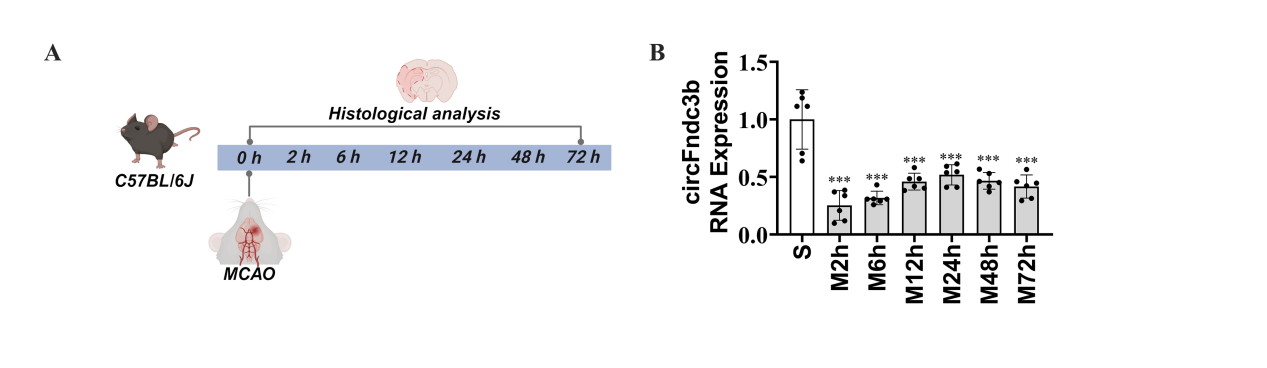


Figure S4. Time course of circFndc3b expression in the peri-infarct cortex.

1. Schematic illustration of the experimental procedures, created with Biorender. (B) Time course of circFndc3b expression in the peri-infarct cortex (n = 6). Results are represented as means ± SD. **P* < 0.05, ***P* < 0.01, ****P* < 0.001. For the statistical analysis of Figure S4A, a one-way ANOVA followed by Tukey’s post hoc test was conducted.


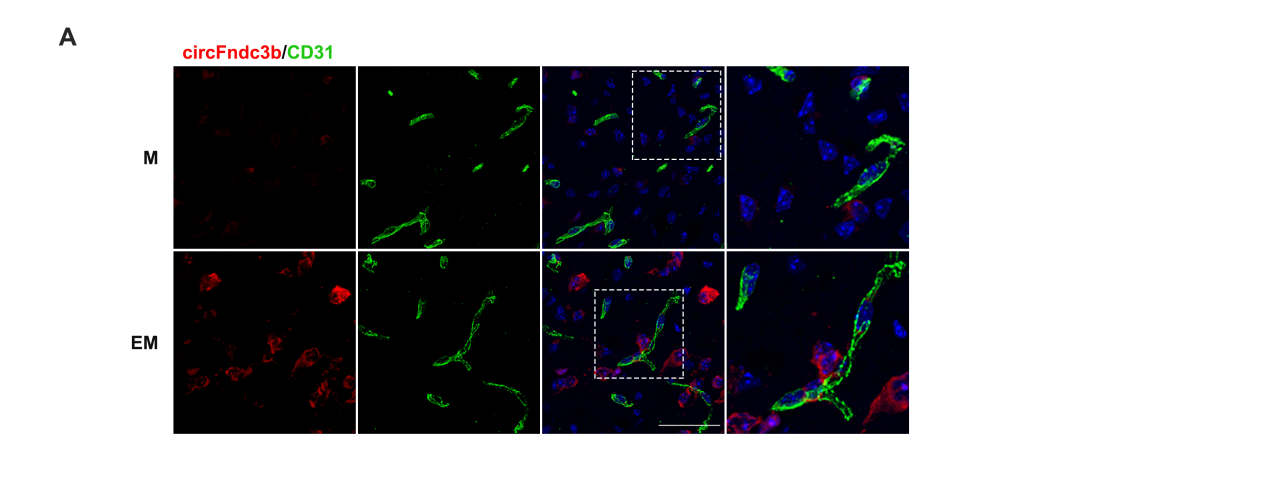


Figure S5. CircFndc3b exhibits minimal colocalization with endothelial cells.

(A) Fluorescence in situ hybridization images using junction-specific probes for circFndc3b show its distribution in endothelial cells (CD31+) within the peri-infarct cortex (n = 3, scale bar = 50 μm).


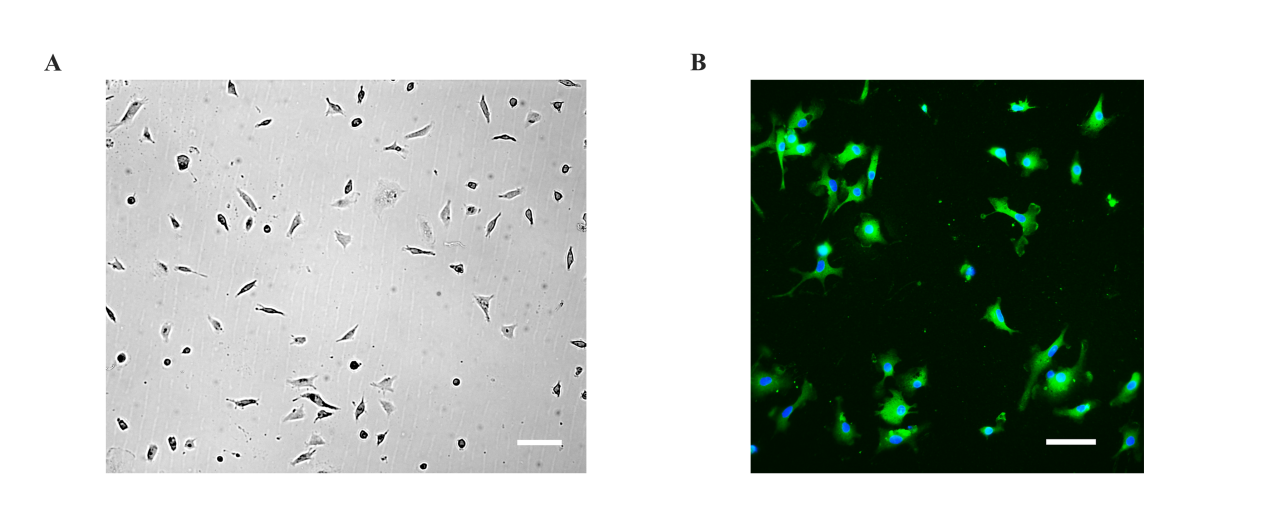


Figure S6. Identification of mouse primary microglia.

1. Representative images of primary microglia by light microscopy (n = 3, Scale bar = 50 μm). (B) Representative images of the immunofluorescence staining of Iba1 in primary MG (n = 3, Scale bar = 50 μm).


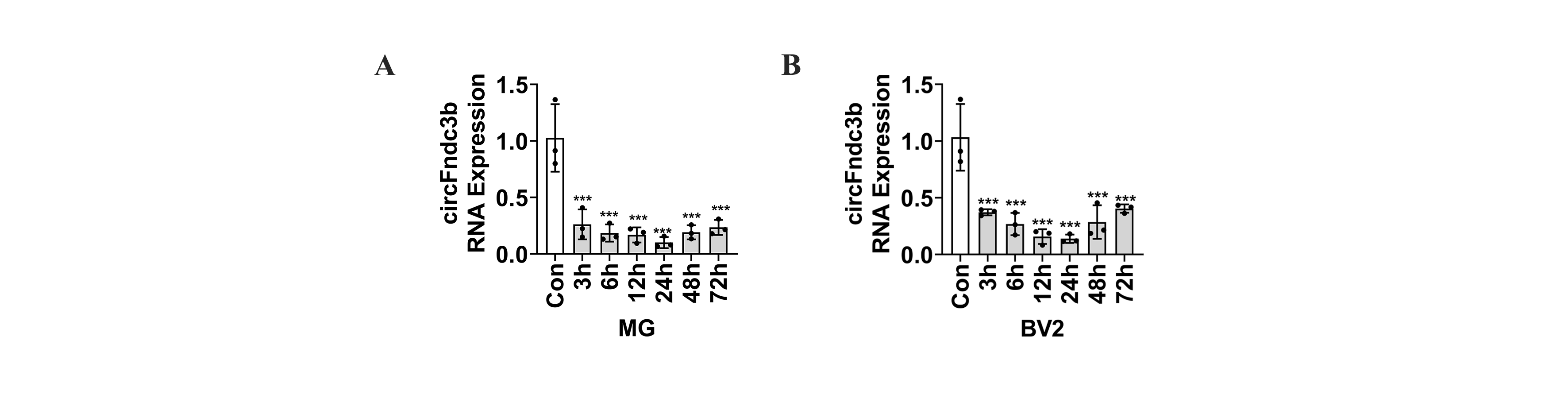


Figure S7. Time course of circFndc3b expression in the primary microglia and BV2 cells after OGD/R.

1. Time course of circFndc3b expression in primary microglia after OGD/R (n = 3). (B) Time course of circFndc3b expression in BV2 cells after OGD/R (n = 3). Data are presented as mean ± SD. **P* < 0.05, ***P* < 0.01, ****P* < 0.001. For the statistical analysis of Figure A–B, a one-way ANOVA followed by Tukey’s post hoc test was conducted.


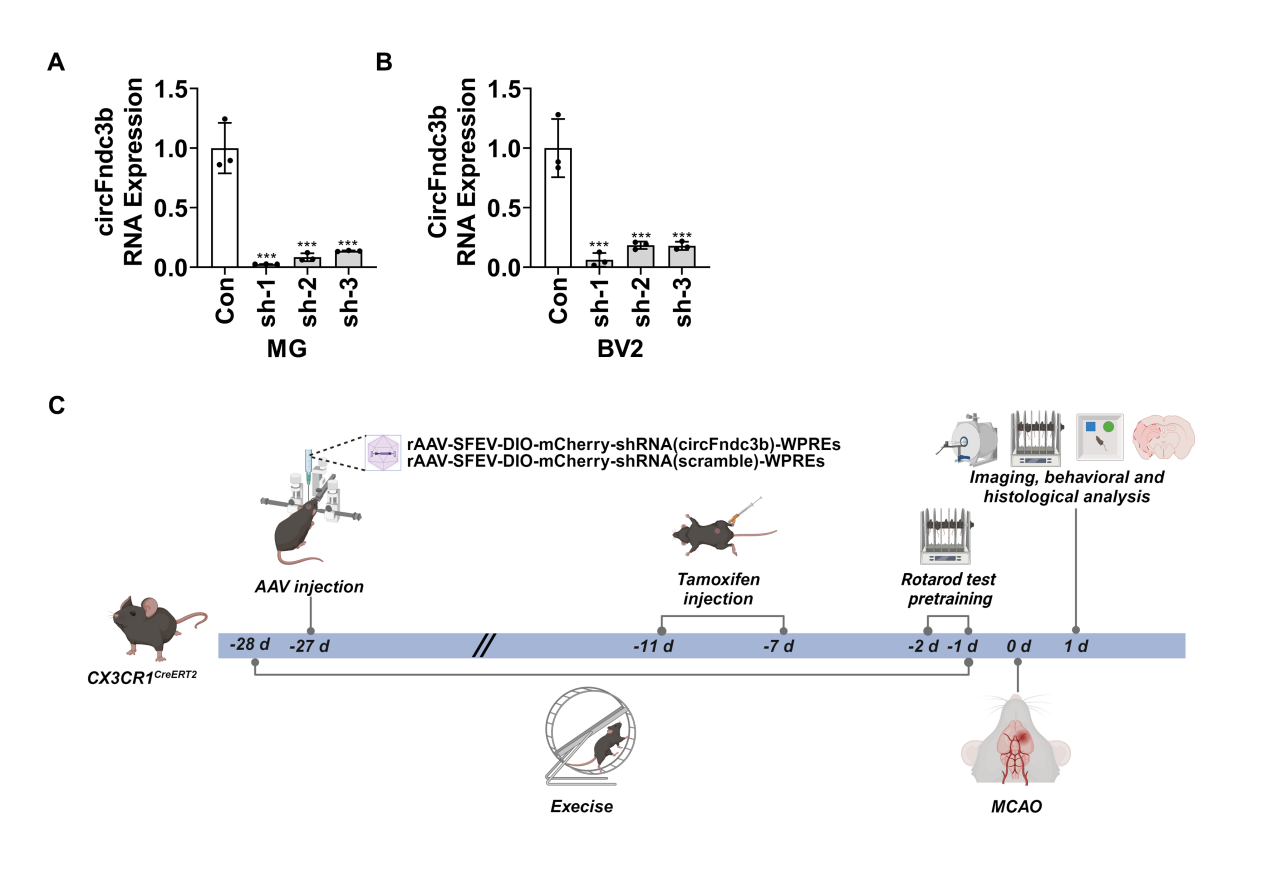


Figure S8. Silencing microglial circFndc3b *in vitro* and *in vivo*.

(A) The silencing efficiency of circFndc3b shRNA in primary microglia (n = 3). (B) The silencing efficiency of circFndc3b shRNA in BV2 cells. (C) Schematic diagram about experimental procedures, created with Biorender. Data are presented as mean ± SD. **P* < 0.05, ***P* < 0.01, ****P* < 0.001. For the statistical analysis of Figure A–B, a one-way ANOVA followed by Tukey’s post hoc test was conducted.


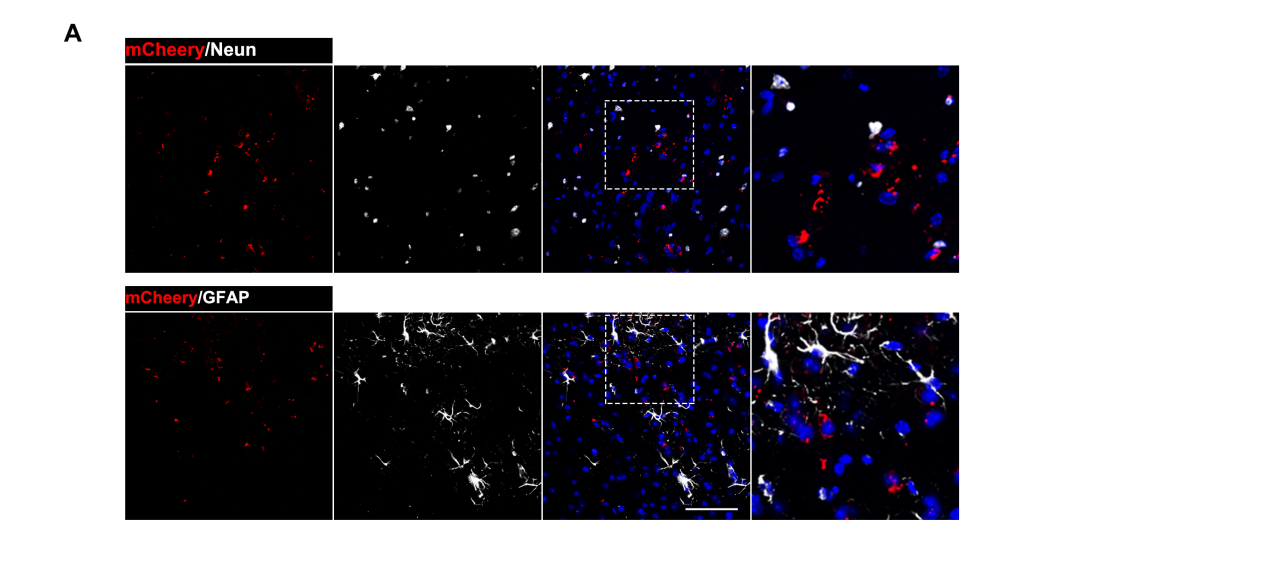


Figure S9. Validation of Cre recombination-dependent virus in neurons (Neun+) and astrocytes (GFAP+).

(A) Representative immunofluorescence images of mCherry+ cells stained with Neun and GFAP (n = 3, Scale bar = 50 μm).


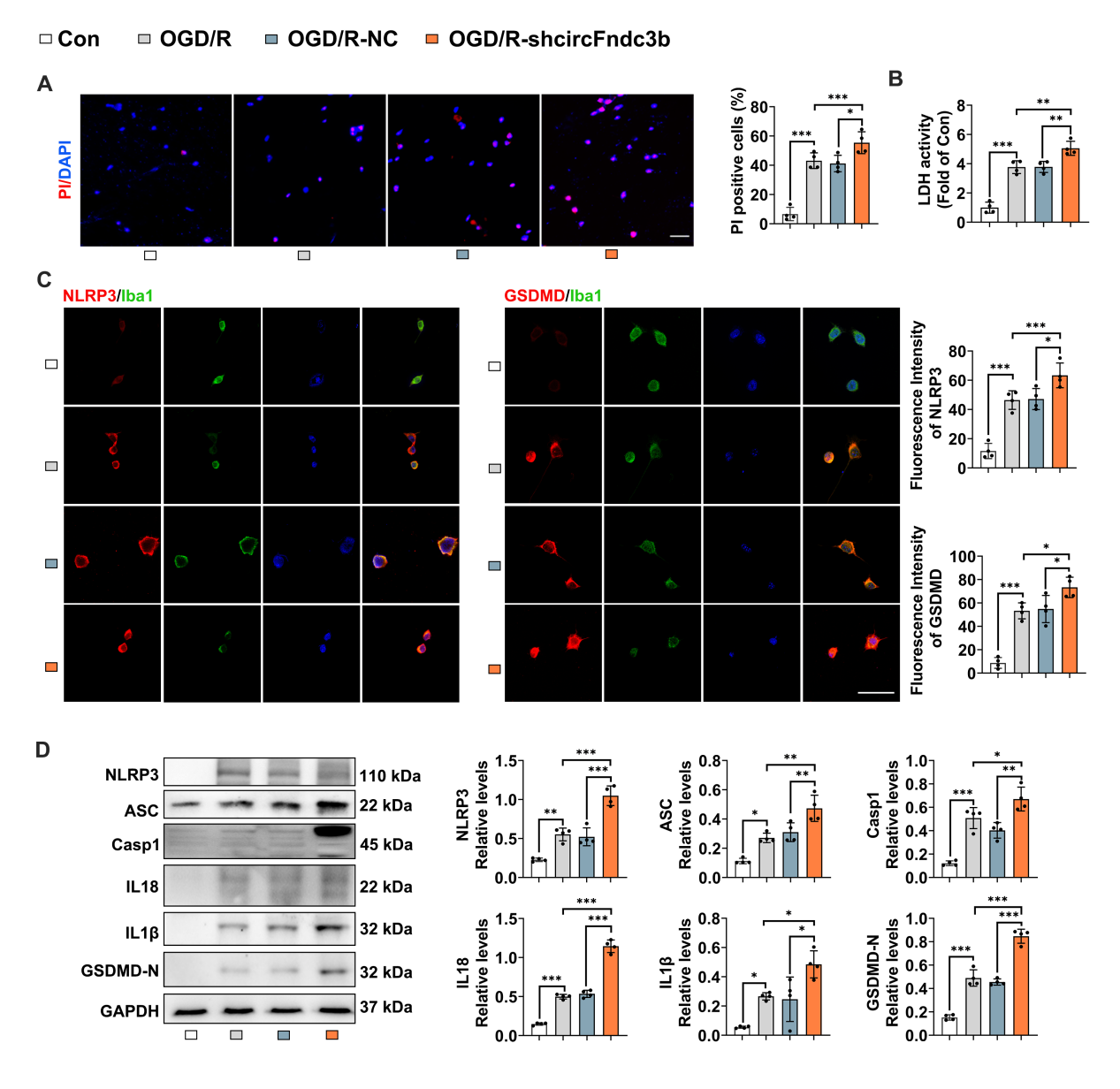


Figure S10. CircFndc3b silencing in BV2 cells aggravated NLRP3 inflammasome-mediated pyoptosis after OGD/R.

1. Representative fluorescence images and the percentage of PI positive cells in BV2 cells inhibiting circFndc3b or negative control and subjected to OGD/R (n = 4, Scales bar = 50 μm). (B) The LDH activity in BV2 cells inhibiting circFndc3b or negative control after OGD/R (n = 4). (C) Representative immunofluorescence images of NLRP3/Iba1 and GSDMD/Iba1 staining in BV2 cells inhibiting circFndc3b or negative control and after OGD/R (n = 4, Scale bar = 50 μm). (D) Western blot analysis of NLRP3, ASC, Casp1, IL18, IL1β, and GSDMD-N expression in BV2 cells inhibiting circFndc3b or negative control after OGD/R (n = 4). Results are represented as means ± SD. **P* < 0.05, ***P* < 0.01, ****P* < 0.001. For the statistical analysis of Figure A–D, a one-way ANOVA followed by Tukey’s post hoc test was conducted.


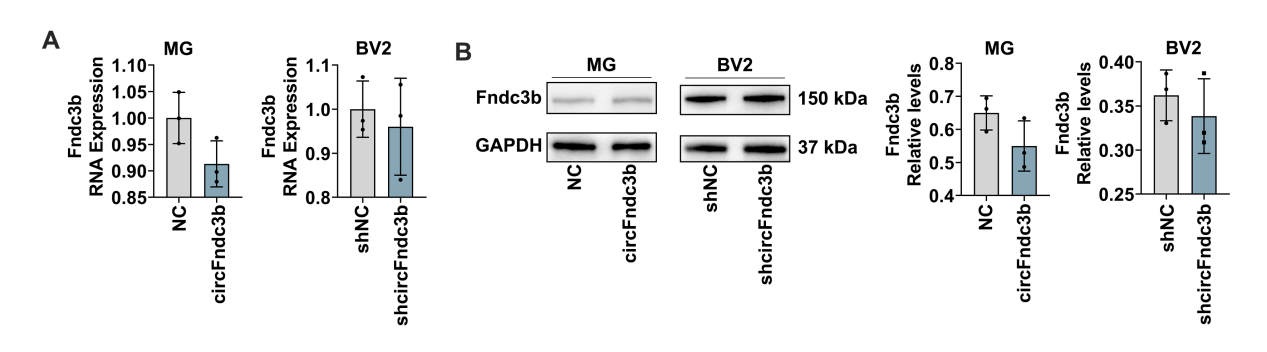


Figure S11. Fndc3b expression in primary microglia and BV2 cells following overexpression or knockdown of circFndc3b.

1. RT-qPCR analysis showing the level of Fndc3b expression in primary microglia and BV2 cells following overexpression or knockdown of circFndc3b (n = 3). (B) Western blotting analysis showing the level of Fndc3b expression in primary microglia and BV2 cells following overexpression or knockdown of circFndc3b (n = 3). Results are represented as means ± SD. **P* < 0.05, ***P* < 0.01, ****P* < 0.001. For the statistical analysis of Figure A–B, an unpaired two-tailed Student’s t-test was performed.


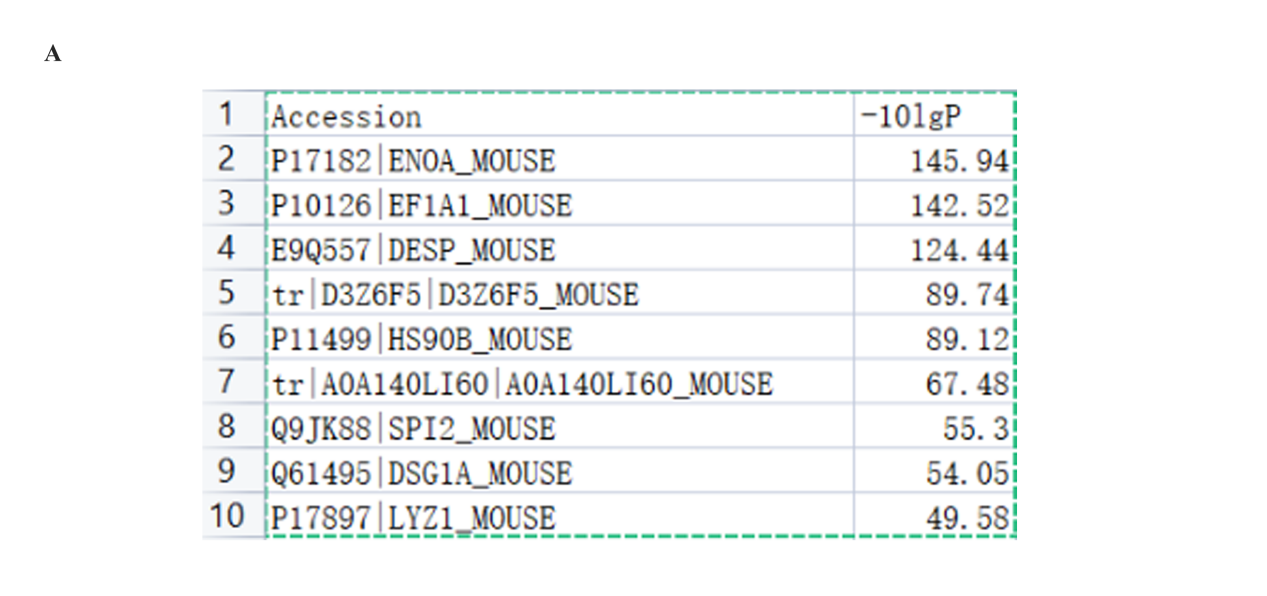


Figure S12. Identification of circFndc3b binding protein using LC-MS.

1. Identification of circFndc3b binding protein using LC-MS ranked top 9.


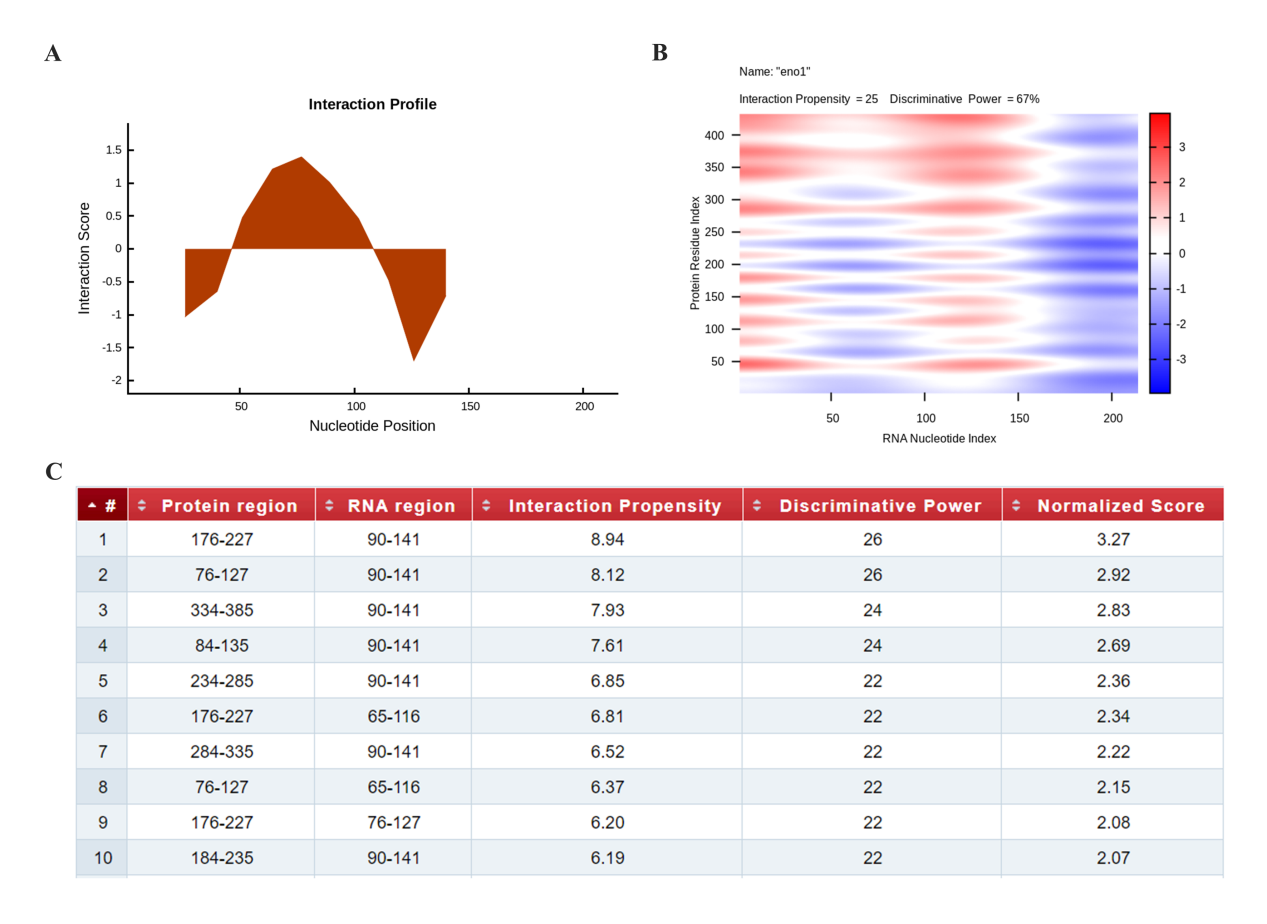


Figure S13. CatRAPID Algorithm predicts the interaction of ENO1-circFndc3b.


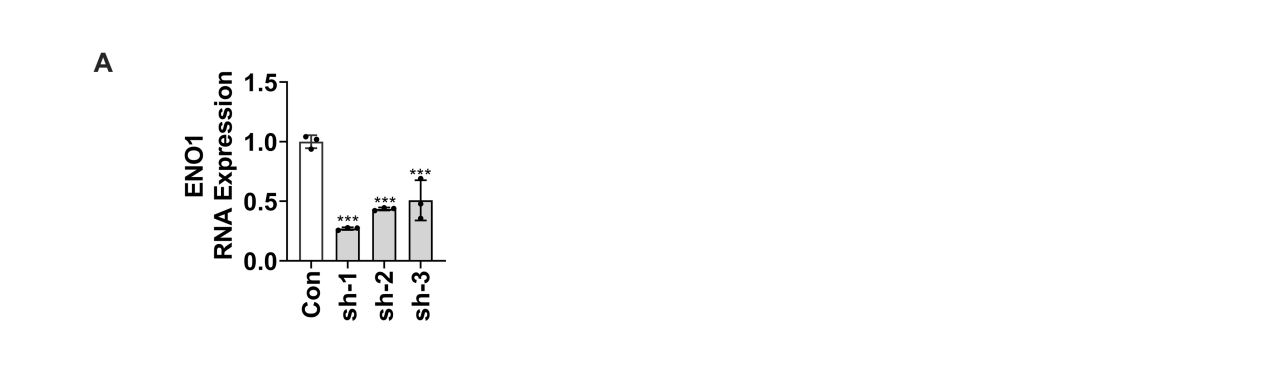


Figure S14. Silencing microglial ENO1 *in vitro*.

(A) The silencing efficiency of ENO1 shRNA in BV2 cells. Data are presented as mean ± SD. **P* < 0.05, ***P* < 0.01, ****P* < 0.001. For the statistical analysis of Figure A, a one-way ANOVA followed by Tukey’s post hoc test was conducted.


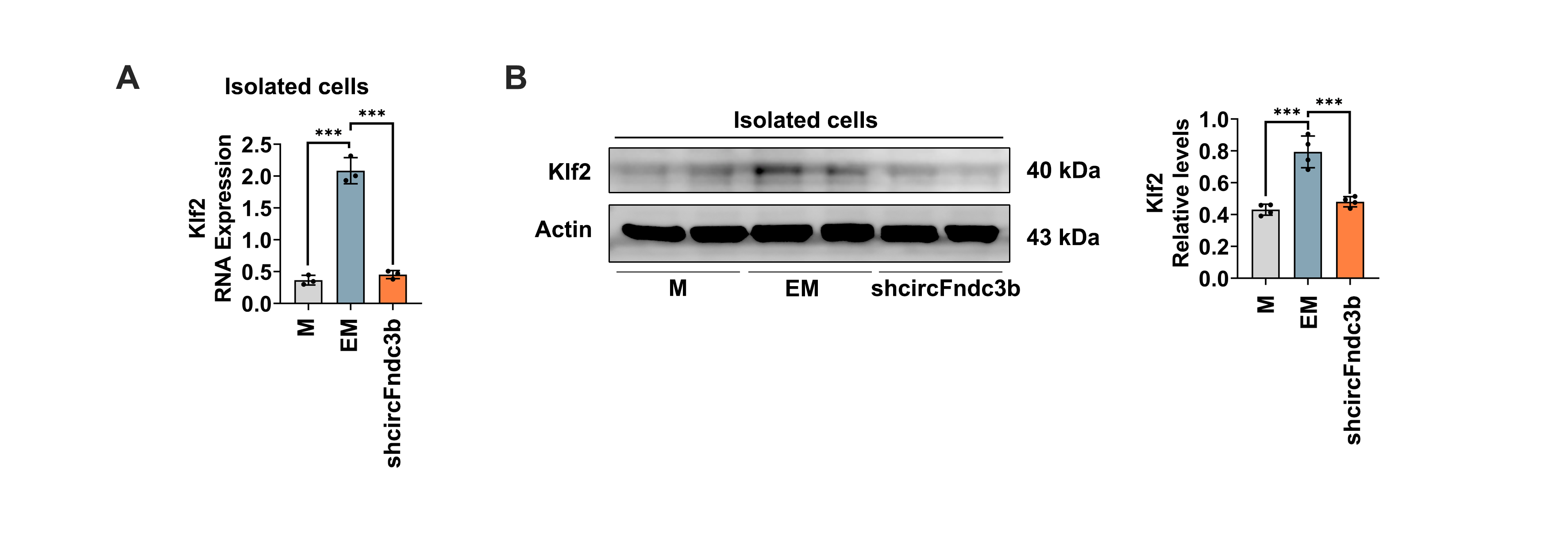


Figure S15. Klf2 expression in isolated microglia/macrophages from the peri-infarct cortex.

1. RT-qPCR analysis showing the level of Klf2 expression (n = 3). (B) Western blotting analysis showing the level of Klf2 expression (n = 4). Data are presented as mean ± SD. **P* < 0.05, ***P* < 0.01, ****P* < 0.001. For the statistical analysis of Figure A–B, a one-way ANOVA followed by Tukey’s post hoc test was conducted.


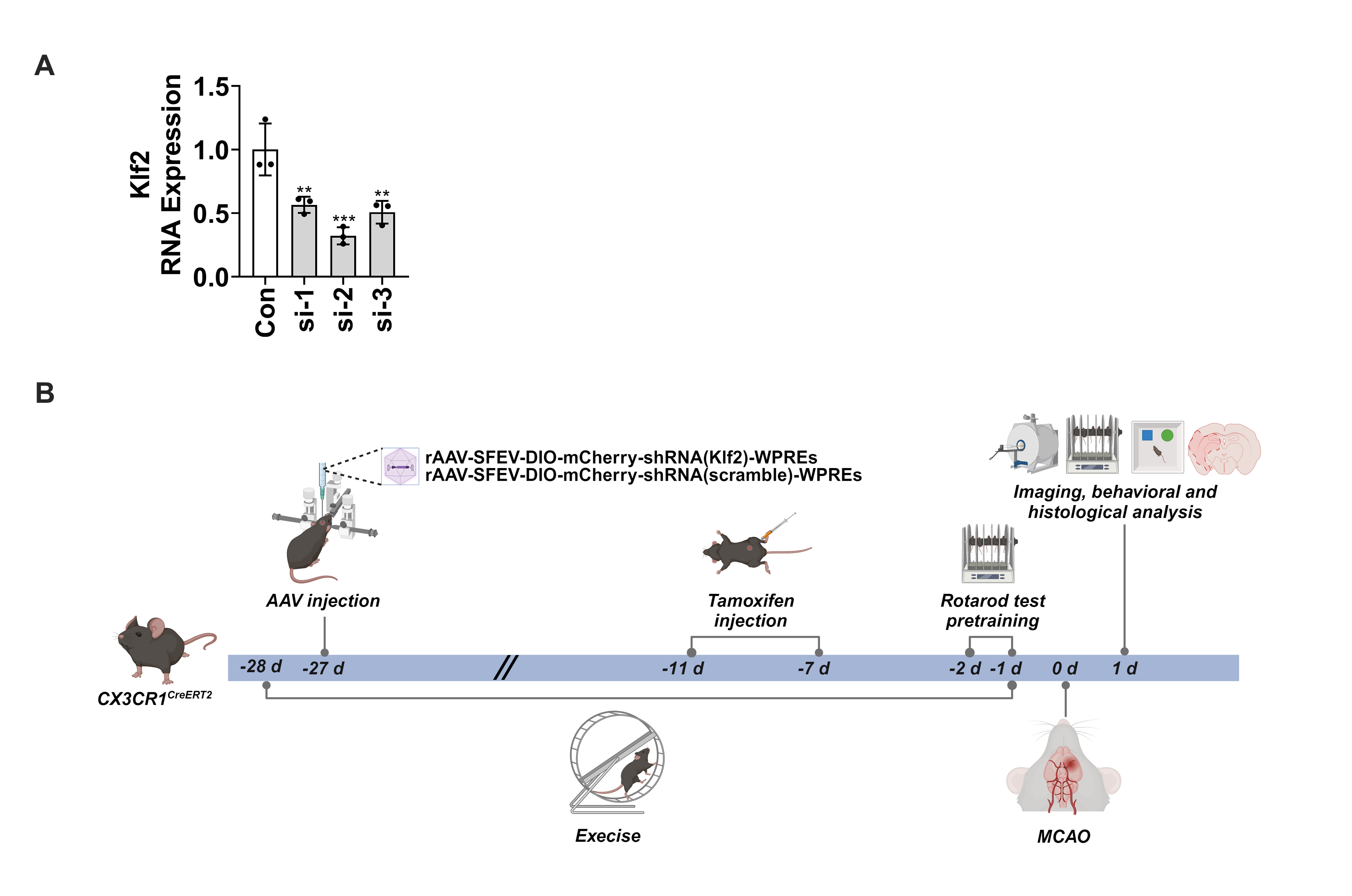


Figure S16. Silencing microglial Klf2 in vitro and in vivo.

(A) The silencing efficiency of Klf2 siRNA in BV2 cells. (B) Schematic diagram about experimental procedures, created with Biorender. Data are presented as mean ± SD. **P* < 0.05, ***P* < 0.01, ****P* < 0.001. For the statistical analysis of Figure A, a one-way ANOVA followed by Tukey’ s post hoc test was conducted.

Supplementary tables

**Table S1. Top 20 differentially expressed circRNAs between M and EM groups**

| Chr_Start_End_Strand | circbaseID |
| --- | --- |
| chr17_81647809_81649638_- | mmu_circ_0000823 |
| chr3_95501347_95507018_+ |  |
| chr14_86828992_86910123_- |  |
| chr3_27620605_27643089_- | mmu_circ_0001113 |
| chr8_56564558_56569410_- |  |
| chr17_10273947_10282981_- | mmu_circ_0006533 |
| chr13_44970046_44996010_- |  |
| chr13_64142208_64145680_- |  |
| chr14_52181196_52181536_- |  |
| chr2_24631899_24639210_- | mmu_circ_0000990 |
| chr12_100446860_100495440_- | mmu_circ_0003641 |
| chr4_9630773_9639347_- | mmu_circ_0011874 |
| chr16_58424561_58433463_+ | mmu_circ_0000693 |
| chr13_63061035_63068285_+ | mmu_circ_0000470 |
| chr17_39845120_39845378_+ | mmu_circ_0000775 |
| chr13_98870240_98884664_- |  |
| chr13_9560671_9576918_+ | mmu_circ_0004907 |
| chr2_91805024_91825352_+ | mmu_circ_0001046 |
| chr10_67034599_67039577_- | mmu_circ_0002360 |

**Table S2. SiRNA/shRNA targeting sequences**

| Mmu_circ_0001113 | sh-1 | CAGTGCATCCAAGGTAGCAGG |
| --- | --- | --- |
|  | sh-2 | GCATCCAAGGTAGCAGGTTGA |
|  | sh-3 | CTTCAGTGCATCCAAGGTAGC |
| ENO1 | sh-1 | CCCGGCTTTCAATGTGATCAA |
|  | sh-2 | CGGCACAGAGAATAAATCTAA |
|  | sh-3 | GGAGCAGAGGTTTACCACAAC |
| Klf2 | si-1 | GCACGGAUGAGGACCUAAATT |
|  | si-2 | CCUUAUCAUUGCAACUGGGAATT |
|  | si-3 | GCACAUGAAGCGACACAUGUATT |
| FUS | si-1 | GCAGGGAGAGGCCATATTA |
|  | si-2 | TCGCAGGGAGAGGCCATAT |
|  | si-3 | GGGCGAGCACAGACAGGAT |

**Table S3. 49 unique proteins interacted with circFndc3b detected by the LC-MS technique**

| Accession | -10lgP | Accession | -10lgP | Accession | -10lgP |
| --- | --- | --- | --- | --- | --- |
| P17182\|ENOA_MOUSE | 145.94 | P63017\|HSP7C_MOUSE | 34.93 | tr\|A0A1B0GT42\|A0A1B0GT42_MOUSE | 23.9 |
| P10126\|EF1A1_MOUSE | 142.52 | P38647\|GRP75_MOUSE | 34.58 | Q7TNL3\|STK40_MOUSE | 22.61 |
| E9Q557\|DESP_MOUSE | 124.44 | P59034\|LRRC3_MOUSE | 34.09 | Q01755\|TCP11_MOUSE | 22.3 |
| tr\|D3Z6F5\|D3Z6F5_MOUSE | 89.74 | tr\|A0A0G2JFH2\|A0A0G2JFH2_MOUSE | 33.88 | tr\|G3X9M2\|G3X9M2_MOUSE | 22.26 |
| P11499\|HS90B_MOUSE | 89.12 | tr\|A0A0U1RNJ1\|A0A0U1RNJ1_MOUSE | 32.83 | P58854\|GCP3_MOUSE | 21.53 |
| tr\|A0A140LI60\|A0A140LI60_MOUSE | 67.48 | tr\|G5E8G6\|G5E8G6_MOUSE | 30.26 | Q45VK7\|DYHC2_MOUSE | 21.53 |
| Q9JK88\|SPI2_MOUSE | 55.3 | tr\|G5E8Z3\|G5E8Z3_MOUSE | 30.03 | Q0VGT4\|ZGRF1_MOUSE | 21.26 |
| Q61495\|DSG1A_MOUSE | 54.05 | Q80Y56\|RBNS5_MOUSE | 29.19 | tr\|Q6XBG2\|Q6XBG2_MOUSE | 21.18 |
| P17897\|LYZ1_MOUSE | 49.58 | Q8BY98\|SWAHD_MOUSE | 28.24 | tr\|A2AQ99\|A2AQ99_MOUSE | 21.07 |
| P10639\|THIO_MOUSE | 48.78 | Q3TRJ4\|K1C26_MOUSE | 28.05 | tr\|F6QGA9\|F6QGA9_MOUSE | 21.06 |
| tr\|A0A087WQS9\|A0A087WQS9_MOUSE | 46.48 | tr\|F6YNQ1\|F6YNQ1_MOUSE | 27.24 | A2A891\|CMTA1_MOUSE | 20.7 |
| tr\|E9Q397\|E9Q397_MOUSE | 42.87 | Q9QXZ9\|OPN4_MOUSE | 26 | P70388\|RAD50_MOUSE | 20.49 |
| P56480\|ATPB_MOUSE | 41.86 | Q8CFS6\|KCNV2_MOUSE | 25.23 | Q06649\|3BP2_MOUSE | 20.29 |
| P09411\|PGK1_MOUSE | 41.37 | P09405\|NUCL_MOUSE | 24.89 | Q60813\|ADM1A_MOUSE | 20.27 |
| P62918\|RL8_MOUSE | 40.95 | tr\|F6SH36\|F6SH36_MOUSE | 24.88 | P42703\|LIFR_MOUSE | 20.01 |
| O70250\|PGAM2_MOUSE | 38.3 | Q9QYR6\|MAP1A_MOUSE | 24.51 | tr\|E9Q7N9\|E9Q7N9_MOUSE | 36.97 |
| tr\|A0A140LIN9\|A0A140LIN9_MOUSE | 24.39 |  |  |  |  |

Table S4. 80 differentially expressed genes downregulated by shcircFndc3b that overlapped with ENO1 Clip-seq data from RNAct

| Klf2 | Egr3 | Hexdc | Evl | Ctage5 | Phf14 | Wnt5b | Mppe1 |
| --- | --- | --- | --- | --- | --- | --- | --- |
| Ccdc142 | Lrrc14 | Kcnk2 | Rbm45 | Tirap | Fancf | Dgat2 | Dyrk1b |
| Antxr1 | Siah1b | St7 | Rab43 | Fus | Dhrs13 | Lemd3 | 2700081O15Rik |
| Paqr3 | Wtap | Marcksl1 | Dusp1 | Smox | Btbd6 | Slc10a3 | Tmbim4 |
| Agap2 | Adat3 | Frat1 | Rhob | Tmcc1 | Akt1s1 | Rnf149 | Orai1 |
| Agpat2 | Egln2 | Mocs3 | Fam122a | Zdhhc5 | Rbm34 | Gse1 | Mecp2 |
| Zfp3 | Smad6 | Dhrs11 | Zfp703 | Doc2a | Dmrta2 | Lipt2 | Txnl4a |
| Nt5m | Hira | Phf10 | Smad7 | Spata1 | Runx3 | Arl2bp | Mfsd5 |
| Peli2 | Mypop | Rcor2 | Rcan1 | Cacng8 | Pcf11 | Gas2l1 | H1f0 |
| Pom121 | Elf2 | Slc1a5 | Gemin7 | Cd63 | Cebpb | Cebpa | Dusp5 |

**Table S5. Primer sequences for RT-qPCR**

| circFndc3bF | CAGCTCAGCAGGTCATTCTT |
| --- | --- |
| circFndc3bR | CTCAACCTGCTACCTTGG |
| ENO1F | GTGTAAGCTGGCCCAATCCA |
| ENO1R | AGGGGCACCAGTCTTGATCT |
| Egr3F | GACAATCTGTACCCCGAGGAGA |
| Egr3R | GTCCATCACATTCTCTGTAGCCA |
| Klf2F | GAGCCTATCTTGCCGTCCTT |
| Klf2R | TGGCTCCTGCACCCTGTA |
| FUSF | ACACACGGAGAGACTCATGC |
| FUSR | CCCAGGCATTCGCCTATACT |
| preFndc3bF | ACCCACCATCCACACTTCATC |
| preFndc3bR | GAGTGTGGCCAGTTTGAACA |
| GAPDH-F | AGGTCGGTGTGAACGGATTTG |
| GAPDH-R | TGTAGACCATGTAGTTGAGGTCA |
| mmu_circ_0000823F  mmu_circ_0000823R | GGCATACTAGAATCCAATCACGCT  GGTTCCCAAATGGGCAAGATC |
| mmu_circ_0006533F  mmu_circ_0006533R | GGAGAATCCTTGGACCTAGAGG  GTAACTGAACAATGGGTCCCACC |
| mmu_circ_0000990F  mmu_circ_0000990R | TGATGTTGAACCTCTTTGTTGCTG  CGATGATGGCGTAGATGAAGAAC |
| Fndc3bF | CACCTCGAGTGACGCAGTTAG |
| Fndc3bR | GTCTCTTCCAACCAGCACCTG |
